# High‐Performance Bi‐Based Catalysts for CO₂ Reduction: In Situ Formation of Bi/Bi₂O₂CO₃ and Enhanced Formate Production

**DOI:** 10.1002/advs.202415616

**Published:** 2025-01-21

**Authors:** Ben Li, Jiadong Chen, Lihua Wang, De Xia, Shanjun Mao, Lingling Xi, Huajie Liu, Sibin Ying, Yong Wang

**Affiliations:** ^1^ Advanced Materials and Catalysis Group ZJU‐Zhejiang Xinhua Low‐Carbon Research Center State Key Laboratory of Clean Energy Utilization Institute of Catalysis Department of Chemistry Zhejiang University Hangzhou 310058 P. R. China; ^2^ College of Chemistry and Molecular Engineering Zhengzhou University Zhengzhou 450001 P.R. China; ^3^ Hunan Provincial Key Laboratory of Environmental Catalysis and Waste Recycling College of Material and Chemical Engineering Hunan Institute of Engineering Xiangtan 411104 P. R. China; ^4^ ZJU‐Zhejiang Xinhua Low‐Carbon Research Center Zhejiang Xinhua Chemical Co., Ltd P. R. China

**Keywords:** Bi/Bi_2_O_2_CO_3_, CO_2_RR, formate, in situ reconstruction, TA

## Abstract

The unavoidable self‐reduction of Bismuth (Bi)‐based catalysts to zero‐valence Bi often results in detrimental adsorption of OCHO^*^, leading to unsatisfactory selectivity of HCOOH in the electroreduction of carbon dioxide (CO_2_). A novel Bi‐tannin (Bi‐TA) complex is developed, which undergoes in situ reconstruction into a Bi/Bi₂O₂CO₃ phase during CO_2_ reduction. This reconstructed catalyst exhibits high activity and selectivity, achieving a Faradaic Efficiency (FE) for formate production exceeding 90%, peaking at 96%. Operando spectroscopic and theoretical analyses reveal that the Bi^δ+^ active site in Bi/Bi₂O₂CO₃ significantly enhances the formation of the OCHO^*^ intermediate, crucial for formate production. The study offers a promising approach to overcoming the limitations of Bi‐based catalysts in CO_2_ reduction to formate.

## Graphical abstract

Catalytically active Bi/Bi_2_O_2_CO_3_ is formed in situ through dynamic restructuring of Bi‐TA complex, the obtained Bi/Bi_2_O_2_CO_3_ leading to an outstanding CO_2_ to formate performance with high activity and selectivity. This study reveals the dynamic structural reconstruction of the electrocatalyst under realistic reaction conditions and offers detailed insights of stable Bi^δ⁺^ site toward CO₂RR.

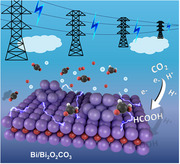

## 1 Introduction

The continuous rise in atmospheric carbon dioxide (CO_2_) levels has prompted an urgent need for effective strategies to mitigate its impact on climate change.^[^ ^1^, ^2^, ^3^, ^4^ ^]^ As a major greenhouse gas, CO_2_ significantly contributes to global warming^[^ ^5^ ^]^ and ocean acidification.^[^ ^6^ ^]^ Traditional methods for reducing CO_2_ emissions, such as carbon capture and storage^[^ ^7^ ^]^ (CCS), face limitations in terms of efficiency, cost, and scalability, making them less than ideal for widespread adoption.^[^ ^8^, ^9^ ^]^ In light of these challenges, converting CO_2_ into valuable chemical products through electrochemical reduction (CO_2_RR) has emerged as a promising alternative.^[^ ^10^ ^]^ This method not only helps address environmental concerns but also opens the possibility of producing valuable chemicals and fuels, contributing to a circular carbon economy by turning a waste product into a resource.^[^ ^11^ ^]^

Over the past decade, substantial progress has been made in the development of electrocatalysts for CO_2_ reduction. These catalysts are often based on metals such as copper,^[^ ^12^ ^]^ silver,^[^ ^13^ ^]^ gold,^[^ ^14^ ^]^ and tin,^[^ ^15^ ^]^ each exhibiting varying degrees of success in reducing CO_2_ into products like carbon monoxide,^[^ ^16^ ^]^ methane,^[^ ^17^ ^]^ ethylene,^[^ ^18^ ^]^ and formate.^[^ ^19^ ^]^ Among these, copper is particularly notable for its ability to produce hydrocarbons and alcohols, though its low selectivity often results in a mixture of products.^[^ ^20^ ^]^ Improving the selectivity of catalysts remains a major hurdle to overcome for practical applications.

Electrochemical CO_2_ reduction to formate has garnered significant attention due to the industrial relevance of formate as both a feedstock for chemical synthesis and a hydrogen carrier in fuel cells.^[^ ^21^ ^]^ Efficient and selective conversion of CO_2_ to formate could transform CO_2_ from an environmental burden into a valuable resource, offering a sustainable method for chemical production and energy storage.^[^ ^22^ ^]^ However, achieving high selectivity and efficiency in CO_2_RR, particularly for formate production, presents several challenges, including the need for catalysts that can operate effectively under practical conditions.^[^ ^23^ ^]^

Bismuth (Bi)‐based catalysts have attracted considerable interest due to their unique ability to selectively reduce CO_2_ to formate. Bismuth is a post‐transition metal with electronic properties that make it highly effective for this reaction. Specifically, its large 6p orbital facilitates the stabilization of the OCHO^*^ intermediate, a crucial step in formate production. Furthermore, Bi is relatively inexpensive, widely available, and non‐toxic, making it a strong candidate for large‐scale applications in CO_2_ reduction.^[^ ^24^ ^]^

Despite these advantages, Bi‐based catalysts are not without their challenges. One of the most significant issues is the competition between the hydrogen evolution reaction^[^ ^25^ ^]^ (HER) and CO_2_RR. During the CO_2_ reduction process, Bi tends to reduce to metallic bismuth, which has a higher activity for HER.^[^ ^26^ ^]^ This shift in catalytic activity reduces the selectivity for formate production, thereby diminishing the overall efficiency of the process.

To address these issues, various strategies have been explored to enhance the performance of Bi‐based catalysts. Nanostructuring, heteroatom doping, and single‐atom alloying have all been investigated to improve activity and selectivity.^[^ ^27^ ^]^ Nanostructuring can increase the surface area and expose more active sites, while heteroatom doping can modify the electronic structure of Bi, potentially improving selectivity by stabilizing key reaction intermediates like OCHO^*^. In addition, alloying Bi with other metals, such as tin^[^ ^28^ ^]^ (Sn) or antimony^[^ ^29^ ^]^ (Sb), has been shown to improve both selectivity and stability. The incorporation of organic ligands or polymers has also been explored as a way to form a protective layer on the catalyst surface, which can suppress HER and enhance CO_2_RR selectivity.

Building on these advancements, the present study introduces a novel approach to overcoming the limitations of Bi‐based catalysts in CO_2_ reduction. This work focuses on the development of a Bi‐tannin (Bi‐TA) complex, which undergoes in situ reconstruction during the CO_2_ reduction reaction, transforming into a Bi/Bi₂O₂CO₃ phase. This reconstructed phase is designed to enhance stability, selectivity, and efficiency, addressing the common problems encountered in previous Bi‐based catalysts. The in situ transformation leverages the unique properties of both Bi and the tannin‐based complex, offering a more robust and selective pathway for formate production by improving OCHO^*^ formation.

## 2 Results and Discussion

### 2.1 Synthesis and Characterization of Catalysts

**Figure 1:**
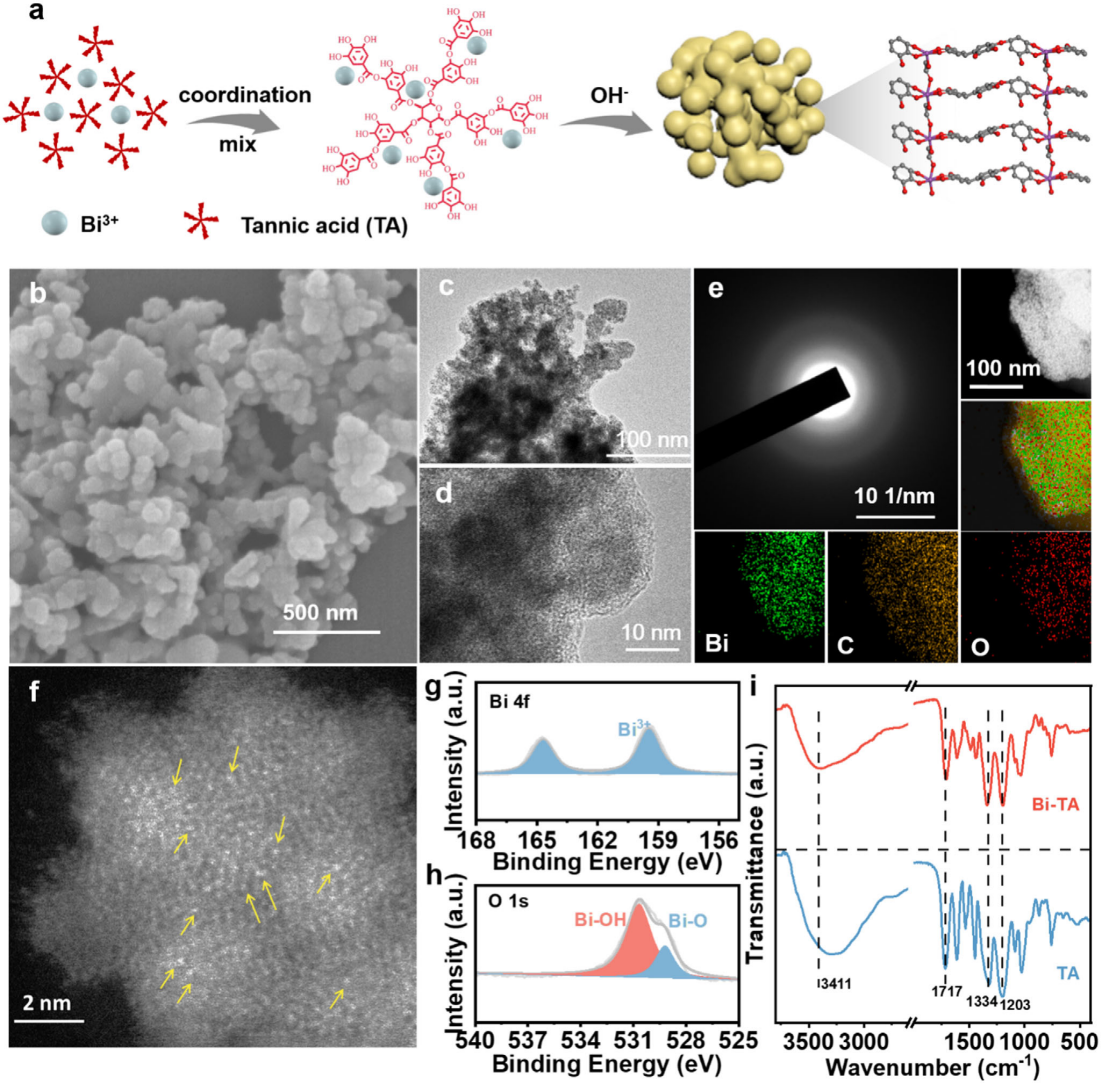
Structural characterizations. a) Schematic illustration for the preparation of the Bi‐TA. b) SEM image of Bi‐TA, c,d) HRTEM images of Bi‐TA, e) HRTEM SAED and EDS of Bi‐TA, f) Atomic‐resolution HAADF‐STEM image of Bi‐TA. g,h) XPS spectra of Bi 4f spectrum and O 1s spectrum of Bi‐TA, i) FT‐IR of Bi‐TA.

The coordination structure of the Bi‐TA complex is formed through the self‐assembly of bismuth ions and tannic acid (TA) under alkaline conditions, resulting in the Bi‐TA complex. Bismuth ions coordinate with catechol groups from different TA molecules, leading to coordination polymerization^[^ ^30^ ^]^ (**Figure** **1**a). Scanning electron microscopy (SEM) and Transmission electron microscopy (TEM) images of the resulting precipitates show an irregular structure (Figure 1b; Figure S1, Supporting Information), while high‐resolution TEM (HRTEM) images (Figure 1c,d) reveal an amorphous nature, as confirmed by the absence of diffuse rings in the selected area electron diffraction (SAED) pattern (Figure 1e). This amorphous structure is further corroborated by the disappearance of characteristic crystalline bismuth peaks in the X‐ray diffraction (XRD) patterns (Figure S3, Supporting Information).

Aberration‐corrected HAADF‐STEM was employed to provide a more detailed view of the Bi‐TA catalyst's morphology. As shown in Figure 1f, bright dots, representing individual Bi atoms (marked with yellow arrows), can be observed in the HADDF‐STEM images. No Bi clusters or nanoparticles are detected across the entire view, indicating the highly uniform dispersion of Bi atoms within the Bi‐TA complex.

Fourier‐transform infrared (FT‐IR) spectroscopy results reveal a blue shift of 80 cm⁻¹ in the O–H stretching frequency of Bi‐TA compared to pure TA, while the C‐O stretching vibration shows little variation. This suggests that Bi^3^⁺ ions predominantly coordinate with the phenolic hydroxyl groups rather than the galloyl groups in TA^[^ ^30^ ^]^ (Figure 1i). Raman spectroscopy further supports this coordination, with the appearance of a peak corresponding to Bi─O modes at 568 cm^−^¹ (Figure S7, Supporting Information). The consistency in the FT‐IR spectra and the detection of Bi in X‐ray photoelectron spectroscopy (XPS) confirms the formation of the Bi‐TA complex (Figure 1g,h). XPS analysis of the Bi 4f spectrum verifies the exclusive presence of Bi^3^⁺ in Bi‐TA, while the O 1s spectrum confirms the existence of Bi─O and Bi─OH bonds.

For comparison, BiOCl was synthesized using the same method, but without the addition of TA, and was characterized in detail (Figures S2–S7, Supporting Information). SEM images show the morphology of the BiOCl nanosheets (Figure S2, Supporting Information), while the XRD pattern displays characteristic peaks at 25.86° (101), 32.49° (110), and 33.45° (102) (Figure S3, Supporting Information), confirming the presence of a tetragonal crystal structure (PDF #06‐0249). HRTEM images and the SAED pattern indicate the exposed crystalline nanosheet structure (Figure S4, Supporting Information). The successful synthesis of crystalline BiOCl without TA highlights TA's crucial role in enhancing the anchoring and dispersion of Bi^3^⁺ ions in the Bi‐TA complex.

### 2.2 Electrochemical CO_2_ Reduction Performance Evaluations

The catalytic properties of the as‐prepared Bi‐TA and BiOCl catalysts for CO₂ reduction were evaluated using a three‐electrode H‐cell with a CO₂‐saturated 0.5 m KHCO₃ electrolyte. Linear‐sweep‐voltammetry (LSV) curves showed a sharp increase in cathodic current for the Bi‐TA catalysts in the CO₂‐saturated electrolyte compared to the Ar atmosphere, indicating catalytic CO₂ reduction (CO₂RR). The product distribution from CO₂ reduction was quantified during constant electrolysis from −0.5 to −1.1 V (vs RHE). Gaseous and liquid products were analyzed using gas chromatography (GC) and ¹H nuclear magnetic resonance (¹H NMR), respectively (Figure S8, Supporting Information). Formate was identified as the only liquid product, with minor amounts of H₂ and CO as byproducts.

**Figure 2:**
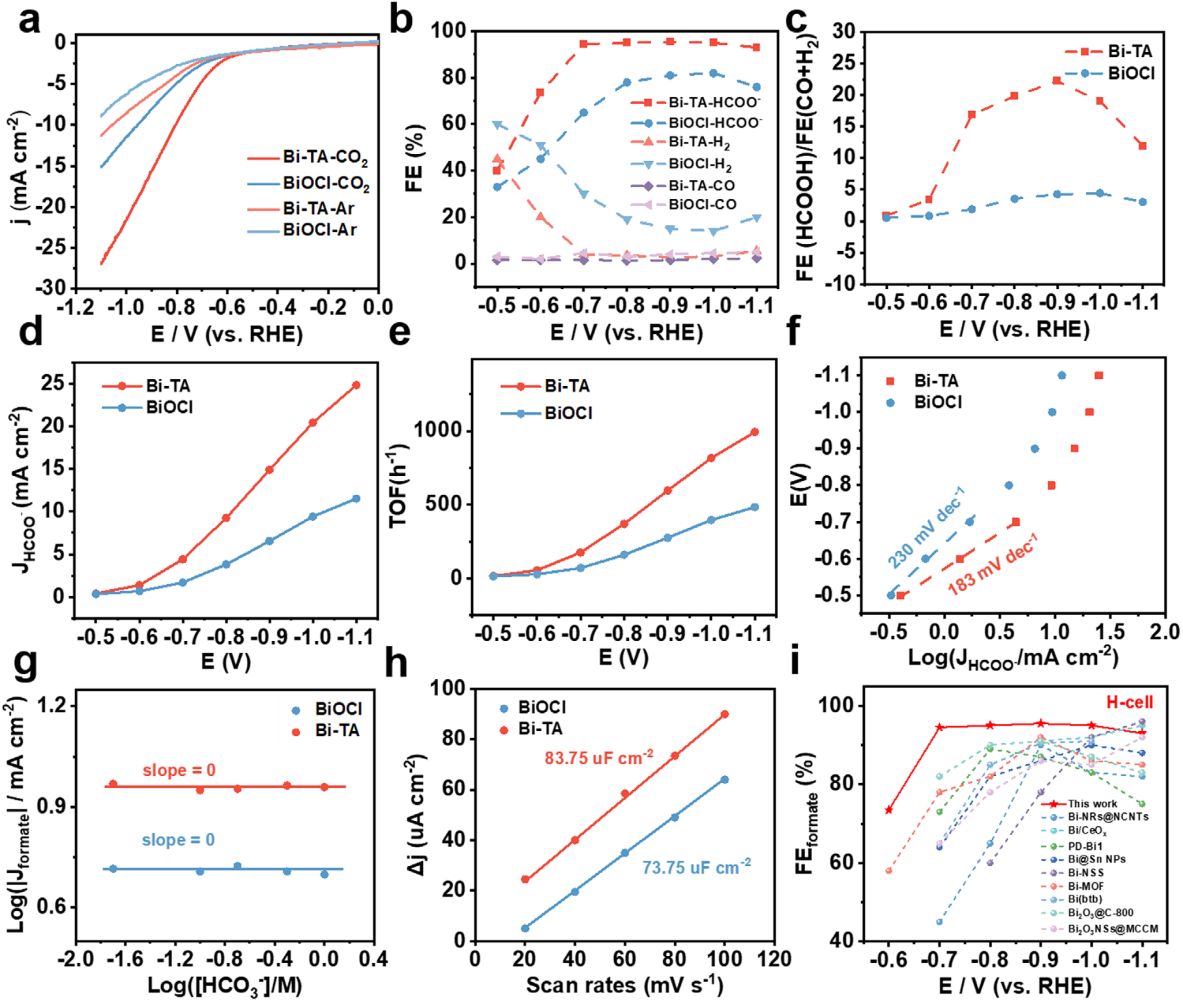
Electrochemical CO_2_ reduction performance in the H‐cell. a) LSV curves in Ar or CO_2_‐saturated 0.5 m KHCO_3_, b) FEs under different working potentials, c) Current density curves of formate formation rate under different working potentials, d) Tafel slope of Bi‐TA and BiOCl, e) Catalytic TOF of formate by Bi‐TA under different working potentials. f) FE ratio of formate over (CO + H_2_), g) Partial current density of formate versus potassium bicarbonate concentration, h) Charging current density differences plotted against scan rates for Bi‐TA and BiOCl, i) Comparison of performances of some typical electrocatalysts reported in the literature for the CO_2_RR to formate.

Bi‐TA catalysts with varying Bi content were synthesized by altering the concentration of BiCl₃ (0.072, 0.048, 0.024, and 0.012 m) during preparation, with the resulting samples denoted as x‐Bi‐TA, where “x” represents the concentration of Bi (x = 0.072, 0.048, 0.024, and 0.012). Among the four catalysts, 0.048‐Bi‐TA (denoted as Bi‐TA) exhibited the best CO₂ reduction performance (**Figure** **2**a; Figure S9, Supporting Information). The formate Faradaic efficiency (FE) for Bi‐TA reached a maximum of 96% at −0.9 V and exceeded 90% over a broad potential range from −0.7 to −1.1 V (vs RHE) (Figure 2b,c). Consequently, Bi‐TA demonstrated a significantly higher formate partial current density (25 mA cm⁻^2^ at −1.1 V) compared to BiOCl (12 mA cm^−^^2^), indicating superior selectivity and activity for CO₂RR (Figure 2d). The turnover frequency (TOF) of Bi‐TA reached a maximum of ≈1000 h^−^¹ at −1.1 V versus RHE, outperforming most reported CO₂‐to‐formate catalysts (Figure 2e; Table S3, Supporting Information).

Tafel plots were employed to assess the reaction kinetics of CO₂ reduction. Bi‐TA and BiOCl exhibited Tafel slopes of 183 and 230 mV dec^−^¹, respectively (Figure 2f), suggesting that the rate‐determining step for both catalysts is the activation of CO₂ to form active intermediates (OCHO^*^ or ^*^COOH) via proton‐coupled electron transfer (CO₂ + e^−^ + H⁺ →OCHO^*^ or ^*^COOH). Additionally, to explore the kinetic dependence on HCO₃^−^ concentration, electrolysis was conducted in CO₂‐saturated electrolytes with HCO₃^−^ concentrations ranging from 0.02 to 1 m. The plot of log(|J_HCOO‐_|) versus log([HCO₃^−^]) revealed an approximately zero‐order dependence on HCO₃^−^ concentration (Figure 2g), indicating that HCO₃^−^ concentration has a negligible effect on the reaction kinetics.^[^ ^31^ ^]^

Electrochemical impedance spectroscopy (EIS) results (Figure S10, Supporting Information) demonstrated that Bi‐TA exhibits significantly lower charge transfer resistance than BiOCl, suggesting enhanced electron transport during CO₂ electroreduction. Furthermore, the double‐layer capacitance (C_dl_), determined by varying the scan rates, was measured to be 83.75 µF cm^−^^2^ for Bi‐TA, higher than that of BiOCl (Figure 2h; Figure S11, Supporting Information), further confirming Bi‐TA's superior surface area and catalytic activity. In summary, the performance of Bi‐TA for CO₂RR was superior to or comparable with previously reported Bi‐based electrocatalysts, as summarized in Figure 2i and Table S2 (Supporting Information). These results collectively indicate Bi‐TA's high selectivity, improved kinetics, and enhanced efficiency for CO₂RR.

**Figure 3:**
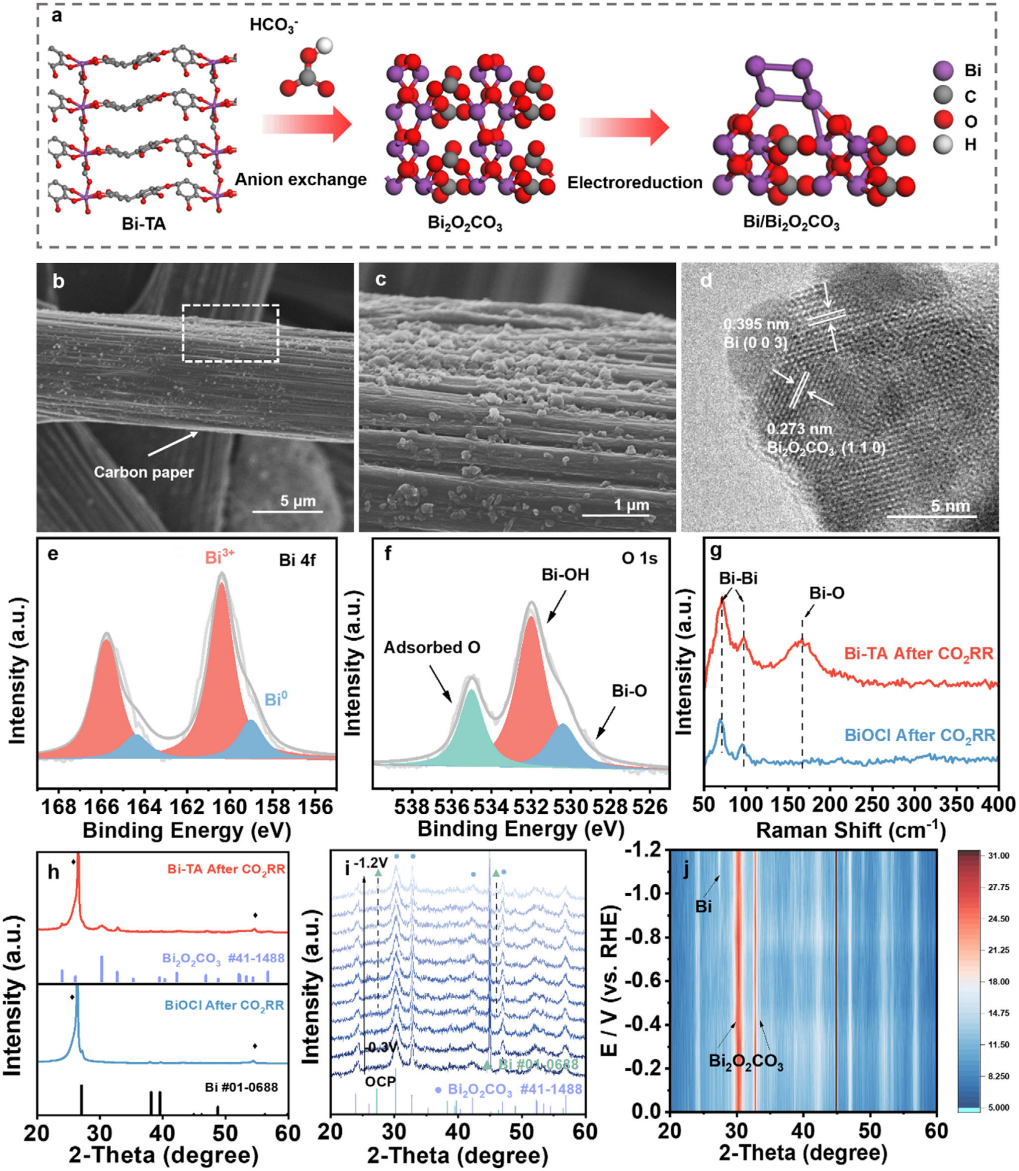
Structural characterizations after CO_2_RR from Bi‐TA to Bi_2_O_2_CO_3_ and Bi/Bi_2_O_2_CO_3_, a) Illustration of the transformation from Bi‐TA to Bi/Bi_2_O_2_CO_3_. b,c) SEM images at different resolutions and d) HRTEM image, e,f) XPS spectra of Bi 4f spectrum and O 1s spectrum of Bi‐TA after CO_2_RR. g) Raman spectra of Bi‐TA and BiOCl after CO_2_RR. h) XRD of Bi‐TA and BiOCl after CO_2_RR. i) In situ XRD of Bi‐TA recorded at various applied potentials (vs RHE) in 0.5 m KHCO_3_ solution and j) Corresponding contour map.

After CO_2_RR, significant changes were observed in the carbon paper electrode background of Bi‐TA‐derived samples, as revealed by SEM measurements (**Figure** **3**b,c), leading to the formation of clusters. The HRTEM image (Figure 3d) showed distinct lattice spacings of 0.395 and 0.273 nm, corresponding to the (003) plane of metallic Bi and the (110) plane of Bi_2_O_2_CO_3_, respectively. XPS analysis was performed to assess the near‐surface composition of the catalyst. The high‐resolution Bi 4f XPS spectra (Figure 3e) indicated the presence of Bi^3+^ (165.9 and 161.3 eV) and Bi^0^ (164.3 and 159.2 eV) components. Additionally, the high‐resolution O 1s XPS spectra were deconvoluted into three main peaks corresponding to Bi–O lattice O, Bi–OH, and surface‐adsorbed O species (Figure 3f).

To further investigate the evolution of our catalyst during CO_2_RR, we conducted Raman spectroscopic measurements. The Raman pattern for the Bi‐TA catalyst revealed three new bands at 168, 95, and 69 cm^−1^ (Figure 3g) after CO_2_RR. The band at 168 cm^−1^ is attributed to a characteristic Raman band of Bi_2_O_2_CO_3_, while the bands at 95 and 69 cm^−1^ are ascribed to Bi–Bi vibrational modes associated with Bi^0^ clusters. This indicates that Bi‐TA contains both Bi^0^ clusters and Bi_2_O_2_CO_3_ after CO_2_RR. For the BiOCl catalyst (Figure S14, Supporting Information), only two Raman bands at 69 and 95 cm^−1^ were observed, attributed to Bi–Bi vibrational modes, suggesting that only Bi^0^ species exist on BiOCl after CO_2_RR. XRD patterns of Bi‐TA confirmed the presence of Bi^0^ and Bi_2_O_2_CO_3_ crystalline phases (Figure 3h), consistent with the HRTEM and XPS results. Conversely, analyses of BiOCl‐derived samples by XRD and HRTEM showed only Bi^0^ (Figure S15, Supporting Information).

Understanding the structural evolution of catalysts during reactions is crucial for studying active phases. We first analyzed the structure of Bi‐TA and found that it may not be theoretically stable due to interactions between Bi^3+^ ions and carboxylates, which correspond to an intermediate acid and a hard base, respectively, according to the Hard–Soft‐Acid–Base (HSAB) principle.^[^ ^32^ ^]^ It is proposed that the Bi─O bond could be disrupted by HCO_3_^−^. To test this hypothesis, Bi‐TA and BiOCl were soaked in a 0.5 m KHCO_3_ solution for 2 h. As shown in Figures S12 and S13 (Supporting Information), the samples reformed and generated new XRD peaks. Comparison with XRD pattern databases indicated that the altered Bi‐TA matched the standard card of Bi_2_O_2_CO_3_. Similar observations were made with BiOCl, confirming that HCO_3_^−^ promotes phase transformation.

To understand the transformation of the catalyst during the CO_2_RR process, we tracked the transformation of Bi‐TA to Bi/Bi_2_O_2_CO_3_ using in situ XRD tests under various reduction potentials. As shown in Figure 3i,j, for Bi‐TA, as the applied potential swept from open circuit potential (OCP) to −1.2 V, Bi_2_O_2_CO_3_ remained the main phase, while metallic Bi diffraction peaks began to appear at −0.7 V. For BiOCl (Figures S16–S18, Supporting Information), the reduction of metallic Bi occurred at −0.5 V, with peak intensity increasing as the potential became more negative, which is more positive compared to Bi‐TA. The Bi_2_O_2_CO_3_ phase gradually disappeared, indicating that the Bi_2_O_2_CO_3_ from Bi‐TA is more stable during anion exchange than Bi_2_O_2_CO_3_. XPS and Raman spectroscopy analyses, consistent with the in situ XRD results, confirmed that the reconstructed Bi_2_O_2_CO_3_ from Bi‐TA via anion exchange is “structurally stable” and can be partly reduced to maintain the Bi_2_O_2_CO_3_ phase, forming the Bi/Bi–O interface responsible for the high activity and selectivity of CO_2_‐to‐formate (Figure 3a).

**Figure 4:**
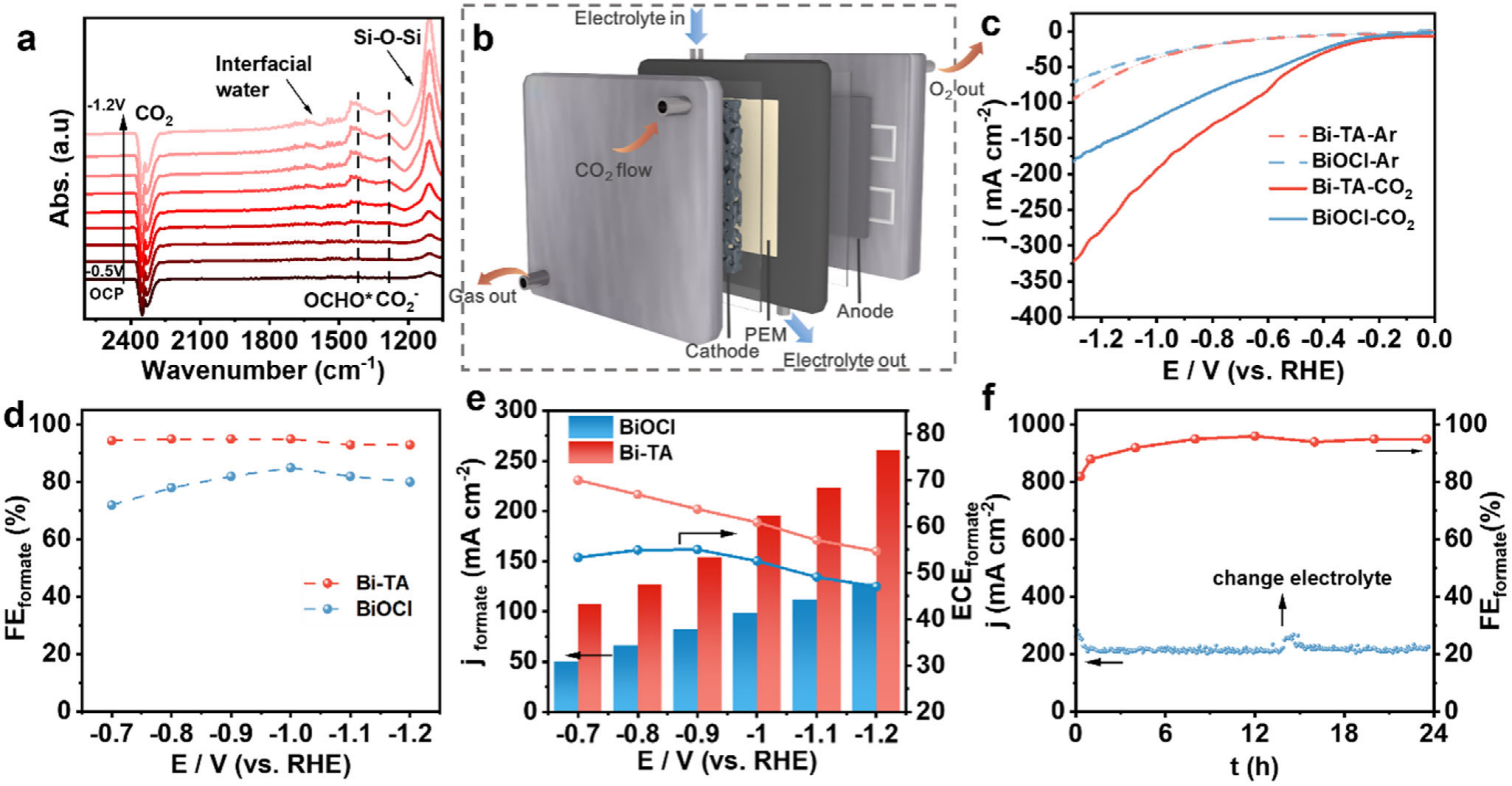
Electrochemical CO_2_ reduction performance in the flow cell. a) Potential‐dependent in situ ATR‐FTIR spectra of Bi‐TA at various applied potentials (vs RHE) in 0.5 m KHCO_3_ solution. b) Schematic diagram of the flow cell. c) LSV curves in Ar or CO_2_‐saturated 1 m KOH. d) HCOOH FEs, e) The half‐cell ECE and formate current densities under different working potentials of Bi‐TA, and f) Stability test of Bi‐TA at −1.0 V versus RHE in 1 m KOH.

In situinfrared absorption spectroscopy with attenuated total reflection mode (ATR‐FTIR) was employed to detect reaction intermediates and further elucidate the mechanisms of CO_2_RR (**Figure** **4**a). The negative peak at 2340 cm^−1^ corresponds to the consumption of CO_2_. As the applied potential increased from −0.5 to −1.2 V, additional peaks at 1286 and 1405 cm^−1^ appeared, which are attributed to the OCHO^*^ intermediate.^[^ ^33^ ^]^ This indicates the efficient proton capture capability of CO_2_^•−^ radicals.

Given the economic importance of formate, a flow‐cell system with 1 m KOH was utilized to enhance CO_2_ mass transfer and assess potential industrial applications. This system demonstrated exceptional formate selectivity across a wide range of potentials. In the flow cell configuration (Figure 4b; Figure S23, Supporting Information) with 1 m KOH, the current density on the Bi‐TA electrode significantly improved, reaching ≈250 mA cm^−2^ at −1.1 V, which represents a substantial increase compared to the H‐cell setup (Figure 4c). Product analysis revealed that the Faradaic efficiencies (FEs) for formate consistently exceeded 92% over a broad potential range from −0.7 to −1.2 V in 1 m KOH (Figure 4d). Moreover, during 24 h of continuous electrolysis, the system sustained a high current density of over 200 mA cm^−2^ with a stable FE exceeding 90% for formate generation (Figure 4f), indicating excellent selectivity at high current densities and promising potential for CO_2_ reduction applications.

Energy conversion efficiency (ECE), defined as the percentage of chemical energy stored in the target product relative to the input electrical energy, is another crucial parameter for the practical application of CO_2_RR.^[^ ^34^ ^]^ The ECE for formate exceeded 50% with a further increase in current density to 250 mA cm^−2^ (Figure 4e). This suggests that a significant portion of electrical energy was utilized for formate production across a broad operational range, providing a positive outlook for the practical application of CO_2_RR in formate formation.

**Figure 5:**
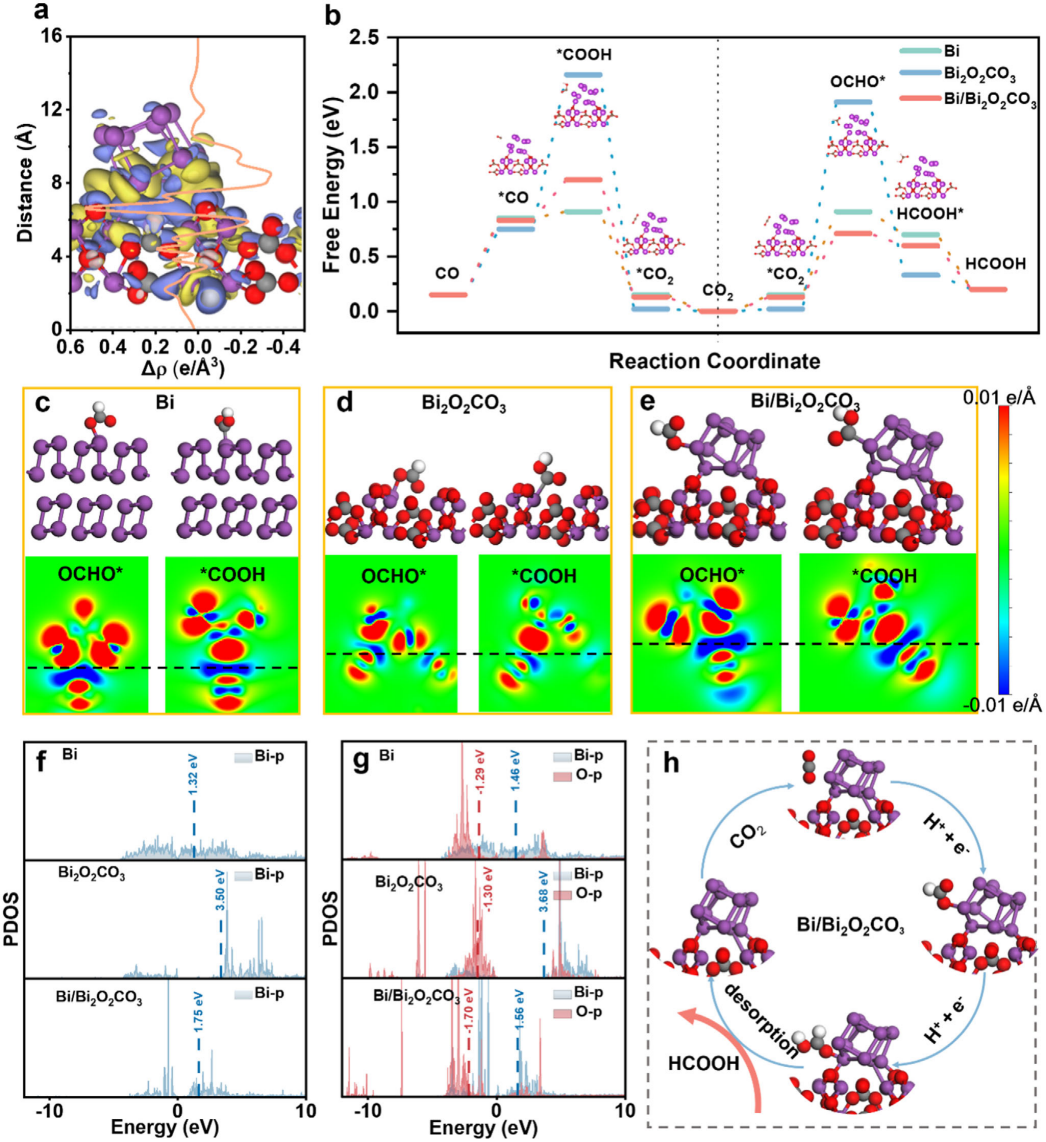
a) The planar average density of charge density difference in the Z direction of Bi/Bi_2_O_2_CO_3_, yellow represents electron aggregation, and blue represents electron loss among them. b) Gibbs free energy diagram for CO_2_RR to HCOOH and CO_2_RR to CO. Optimized configurations of OCHO^*^ and ^*^COOH and the corresponding difference charge densities on c) Bi, d) Bi_2_O_2_CO_3_, and e) Bi/Bi_2_O_2_CO_3_, the dashed lines indicate the positions between the Intermediates and surfaces. f) PDOS of Bi 5p orbitals of Bi, Bi_2_O_2_CO_3,_ and Bi/Bi_2_O_2_CO_3_, g) PDOS of the oxygen atom of the OCHO^*^ intermediate and surface Bi atoms on three models, h) Proposed reaction mechanism for CO_2_ to formate on Bi/Bi_2_O_2_CO_3_.

The intrinsic mechanism for enhancing CO_2_RR to formate was investigated using Density functional theory (DFT) calculations for the reduction of CO_2_ into HCOOH and CO on Bi, Bi_2_O_2_CO_3_, and Bi/Bi_2_O_2_CO_3_. The Bi/Bi_2_O_2_CO_3_ configuration resulted in charge redistribution at the interface, with positively charged Bi atoms at the Bi/Bi_2_O_2_CO_3_ interface (**Figure** **5**a). This charge perturbation plays a crucial role in altering the adsorption behavior of reaction intermediates and influencing the activation and conversion of CO_2_. The Gibbs free energies for CO_2_RR on Bi, Bi_2_O_2_CO_3_, and Bi/Bi_2_O_2_CO_3_ were calculated for the formation of HCOOH and CO (Figure 5b). The optimized adsorption configurations are shown in Figures S19–S21 (Supporting Information).

The energy diagrams indicated that the formation of OCHO^*^ (0.8, 1.85, 0.6 eV), generated by adding the first proton‐electron pair, had a lower Gibbs free energy compared to the formation of ^*^COOH (1.2, 2.1, 0.75 eV) on all three catalysts. This suggests that Bi, Bi_2_O_2_CO_3_, and Bi/Bi_2_O_2_CO_3_ are more selective for CO_2_RR to HCOOH. Notably, the Gibbs free energy at the rate‐determining step (RDS) for HCOOH generation was lower on Bi/Bi_2_O_2_CO_3_ (0.6 eV) compared to Bi_2_O_2_CO_3_ (0.85 eV) and Bi (1.88 eV), indicating enhanced CO_2_RR activity and selectivity.

Charge density differences and Bader charge analysis (Table S6, Supporting Information) for Bi (Figure 5c), Bi_2_O_2_CO_3_ (Figure 5d), and Bi/Bi_2_O_2_CO_3_ (Figure 5e) models with ^*^COOH/OCHO^*^ adsorbates were also examined. Bi/Bi_2_O_2_CO_3_ showed the strongest electronic density interaction with OCHO^*^ among the three models and weaker interaction with ^*^COOH compared to Bi. These observations suggest a favorable environment for formate production.

Furthermore, the outermost valence p orbitals of Bi atoms on Bi, Bi_2_O_2_CO_3_, and Bi/Bi_2_O_2_CO_3_ surface models (without adsorbates) were analyzed, along with the p‐band centers within the overall range of orbitals (Figure 5f). The p‐band center of the Bi/Bi_2_O_2_CO_3_ interface model was found to lie between those of Bi and Bi_2_O_2_CO_3_, indicating a moderate oxide state or electron reservoir. To understand the high performance of the Bi/Bi_2_O_2_CO_3_ surface, we examined the projected density of states (PDOS) based on the electron density and atomic orbital contributions of Bi and O atoms in the adsorbed OCHO^*^ intermediate. Figure 5g shows that Bi/Bi_2_O_2_CO_3_ and pure Bi exhibit significantly larger overlaps and more delocalization between Bi 6p and O 2p compared to Bi_2_O_2_CO_3_, indicating stronger Bi─O bonding in these models.^[^ ^34^ ^]^

Additionally, the DOS at the Fermi energy level (E_f_) provides a rough estimation of electron availability, and the outermost valence p orbital of Bi with the p‐band center was evaluated.^[^ ^35^ ^]^ Notably, compared to pure Bi, the upshifted p‐band centers of Bi on the Bi/Bi_2_O_2_CO_3_ surfaces favor the adsorption of electron‐rich O atoms (electron donors). This trend is further supported by the PDOS of the adsorbed ^*^COOH intermediate, which illustrates electron transfer from ^*^COOH to the unoccupied orbitals of Bi through σ bonding (Figure S22, Supporting Information). Furthermore, Bi/Bi_2_O_2_CO_3_, with downshifted p‐band centers, suppresses σ bonding interactions compared to pure Bi, thereby reducing competitive CO formation.^[^ ^36^ ^]^

Based on the DFT calculations, we propose a plausible reaction mechanism for CO_2_RR on Bi/Bi_2_O_2_CO_3_, as illustrated in Figure 5h. The Bi^3+^ in Bi/Bi_2_O_2_CO_3_ facilitates CO_2_ adsorption, leading to the formation of OCHO^*^. This process is thermodynamically favorable and results in the production of formate.

## 3 Conclusion

The study successfully demonstrates the potential of a Bi‐tannin (Bi‐TA) complex in overcoming the challenges associated with Bi‐based catalysts in CO_2_ reduction. Through in situ reconstruction into a stable Bi/Bi₂O₂CO₃ phase, the catalyst maintains high selectivity and activity for formate production, with a Faradaic Efficiency (FE) exceeding 90%. The presence of Bi^δ+^ active sites plays a crucial role in enhancing the formation of the OCHO^*^ intermediate, essential for efficient CO_2_ reduction. This research provides valuable insights into the design of advanced catalysts for electrochemical CO_2_ reduction, paving the way for more efficient and sustainable industrial applications.

## Conflict of Interest

The authors declare no conflict of interest.

## Supporting information



Supporting Information

## Data Availability

Research data are not shared.
