# Supplementary material for: High‐Performance Bi‐Based Catalysts for CO₂ Reduction: In Situ Formation of Bi/Bi₂O₂CO₃ and Enhanced Formate Production
Source: Adv Sci (Weinh). 2025 Jan 21;12(10):2415616. doi: 10.1002/advs.202415616 (PMC11905003; doi:10.1002/advs.202415616)
Supplement: Supplementary file 1 — Supporting Information [file ADVS-12-2415616-s001.docx]

Supporting Information

**High-Performance Bi-Based Catalysts for CO₂ Reduction: In Situ Formation of Bi/Bi₂O₂CO₃ and Enhanced Formate Production**

*Ben Li^a^, Jiadong Chen^a^, Lihua Wang^a^, De Xia^a^, Shanjun Mao^a^, Lingling Xi^a^, Huajie Liu^c*^, Sibin Ying^d*^*, *Yong Wang^a,b^**

^a^Advanced Materials and Catalysis Group, ZJU-Zhejiang Xinhua Low-Carbon Research Center, State Key Laboratory of Clean Energy Utilization, Institute of Catalysis, Department of Chemistry, Zhejiang University, Hangzhou, 310058, P. R. China

^b^College of Chemistry and Molecular Engineering, Zhengzhou University, Zhengzhou, 450001, P.R. China

^c^Hunan Provincial Key Laboratory of Environmental Catalysis and Waste Recycling, College of Material and Chemical Engineering, Hunan Institute of Engineering, Xiangtan 411104, P. R. China

^d^ZJU-Zhejiang Xinhua Low-Carbon Research Center, Zhejiang Xinhua Chemical Co., Ltd., P.R. China

*Corresponding Author.

E-mail: [chemwy@zju.edu.cn](mailto:chemwy@zju.edu.cn) (Yong Wang)

**Experimental methods**

**Materials**

Tannic acid (TA, >98%), bismuth trichloride (BiCl₃, >99%), thioacetamide, potassium bicarbonate (KHCO₃, >99%), hydrochloric acid (HCl, >98%), potassium hydroxide (KOH, >99%), ethanol (CH₃CH₂OH, >99%), and Nafion solution (5 wt.%) were all of analytical grade and used without further purification.

**Preparation of Bi-TA complex**

Typically, 2×10^-^³ M tannic acid (TA) was dissolved in 25 mL of water and treated with ultrasound for 10 minutes. Then, 20 mL of 0.048 M BiCl₃ solution was added dropwise into 20 mL of water, followed by ultrasonic treatment for an additional 10 minutes. Subsequently, 5 mL of 2×10^-^³ M TA aqueous solution was added dropwise. Afterward, a sufficient amount of 1.0 M KOH solution was added dropwise in sequence under continuous ultrasonic treatment. After 10 minutes, the resulting Bi-TA complex was washed several times with ultrapure water and ethanol, then dried under vacuum. For comparison, a BiOCl sample was prepared using the same method but without the addition of TA.

**Materials characterizations**

Scanning electron microscopy (SEM) was performed using a Hitachi SU8010 microscope. High-resolution transmission electron microscopy (HRTEM), high-angle annular dark-field scanning transmission electron microscopy (HAADF-STEM), and energy-dispersive X-ray spectroscopy (EDS) mapping were carried out on a JEOL JEM-2100F, with spherical aberration correction achieved on a FEI Titan G2 80-200 ChemiSTEM at an acceleration voltage of 200 kV. Powder X-ray diffraction (XRD) patterns were recorded on a Rigaku Ultima IV, operating at 40 kV and 20 mA, using Cu Kα radiation. Inductively coupled plasma-atomic emission spectrometry (ICP-OES) was conducted on a Perkin Elmer Optima OES 800. X-ray photoelectron spectroscopy (XPS) was performed using an Escalab 250Xi spectrometer, and Raman spectra were collected using a LabRam HRUV.

**Electrochemical Measurements**

Electrochemical experiments were conducted using a three-electrode system on a CHI 760E electrochemical workstation (CH Instruments). All polarization curves were recorded without iR compensation. For CO₂ reduction experiments, linear sweep voltammetry (LSV) was performed at a scan rate of 5 mV s⁻¹ in a CO₂-saturated 0.5 M KHCO₃ solution (50 mL), with the electrolyte purged with CO₂ for 30 minutes before measurement. Gas products from CO₂ reduction reactions (CO₂RR) were analyzed using an on-line micro gas chromatograph (GC) equipped with a flame ionization detector (FID) and a thermal conductivity detector (TCD), with Ar (99.999%) serving as the carrier gas. Formate was quantified by nuclear magnetic resonance (NMR) spectroscopy (Agilent DD-600, 600 MHz). The Faradaic efficiency (FE_i_) was calculated using the formula: FE_i_ = Q_i_ / Q_total_ = (n_i_NF) / Q, where n_i_ is the number of moles of a specific product measured by NMR or GC, and Q represents the total charge accumulated during the CO₂ reduction process.

The CO₂ reduction reaction (CO₂RR) performance was first evaluated in a H-type cell with a three-electrode configuration. In this setup, a Pt foil (11 cm²) was used as the counter electrode, an Ag/AgCl reference electrode, and a piece of hydrophobic carbon paper (11 cm²) as the working electrode. For the preparation of the working electrode, catalytic ink was made by dispersing 10 mg of catalyst powder into 990 μL ethanol and 10 μL of 5 wt% Nafion, followed by ultrasonication for at least 1 hour. A 100 μL aliquot of the catalyst ink was applied to the hydrophobic carbon paper and allowed to dry in air, resulting in a catalyst loading of 1 mg cm².

All potentials vs. the saturated calomel electrode (SCE) were converted to the reversible hydrogen electrode (RHE) using the equation: E (vs. RHE) = E (vs. SCE) + E^θ^_SCE_ + 0.0592 pH. Hydrogen evolution reaction (HER) activity measurements were conducted at 25 °C, with LSV curves recorded at a scan rate of 5 mV s⁻¹. Electrochemical impedance spectroscopy (EIS) was measured at an overpotential of 100 mV, over a frequency range from 100,000 Hz to 0.01 Hz, with an amplitude of 5 mV. The electrochemical surface area (ECSA) was evaluated by measuring the electrical double-layer capacitance (C_dl_) of the electrocatalysts using cyclic voltammetry in a non-Faradaic potential window.

Flow cell measurements were conducted using a flow cell reactor with electrocatalyst-loaded gas diffusion electrodes (GDL, 1 mg cm^-^², 2×3 cm²). A platinum sheet and a saturated Ag/AgCl electrode were used as the counter and reference electrodes, respectively, with 1 M KOH as the electrolyte. During the measurements, CO₂ gas was supplied directly to the working electrode at a rate of 20 sccm.

The energy conversion efficiency (ECE_formate_) for CO_2_RR to formate was calculated as follows: ECE_formate_ (%)=(1.23-E_formate_)*FE_formate_ / (1.23-E_app_)*100%. where the kinetic potential E_formate_ for the CO_2_RR to formate is -0.12 V vs. RHE, and E_app_ represent the applied potential (vs RHE) in the flow cell.

**ATR-FTIR Testing**

Fourier transform infrared spectra were collected on a Nicolet iS50 FT-IR Spectrometer. The silicon prism was first coated with a gold film by chemical deposition. The catalytic ink was prepared by dispersing 10 mg of catalyst powder into 990 μL of ethanol and 10 μL of 5 wt% Nafion, followed by ultrasonication for at least 1 hour. A 20 μL aliquot of the ink was deposited onto the gold film. The treated silicon prism, Ag/AgCl electrode, and Pt plate served as the working electrode, reference electrode, and counter electrode, respectively. A 0.5 M KHCO₃ solution purged with CO₂ was used as the electrolyte. Chronoamperometric tests were conducted from -0.4 to -1.2 V vs. RHE on a CHI 760E electrochemical workstation, accompanied by spectrum collection. All spectra were collected after background subtraction.

**Theoretical calculation methods**

Density functional theory (DFT) calculations were performed using the Vienna Ab initio Simulation Package (VASP), employing the plane-wave technique and the Perdew-Burke-Ernzerhof (PBE) exchange-correlation functional. The DFT-D3 method was applied to account for van der Waals interactions. The projector augmented wave (PAW) method was used to describe electron-ion interactions, with a plane-wave cutoff energy of 400 eV. The Bi (001) and Bi₂O₂CO₃ (110) surfaces were chosen for the calculations. A 3×3×1 and 1×1×1 Monkhorst-Pack k-point mesh were used for electronic structure calculations and structural optimizations. All atoms in the supercell were allowed to relax until the force on each atom was less than 0.02 eV/Å.

The adsorption energy of species X (where X represents CO₂, HCOOH, CO, H, OCHO, or HCOO) on the catalyst surface was calculated using the equation: ΔEₓ = Eₓ_/surf_ - Eₓ - E_surf_, where Eₓ_/surf_ is the total energy of the surface with X adsorbed, Eₓ is the energy of the adsorbate X, and E_surf_ is the energy of the clean surface of the catalyst. Thermodynamic free energies were calculated as G = E_DFT_ + E_ZPE_ - TS, where E_DFT_ is the DFT ground state energy, E_ZPE_ is the zero-point energy, and S is the entropy. VASP was subsequently used to optimize the adsorption configurations.

The d-band center was calculated using the formula ε_d_ =∫E • ρ(E) dE /∫ρ(E) dE, where E is the energy relative to the Fermi level (E_f_), and ρ(E) is the density of states.


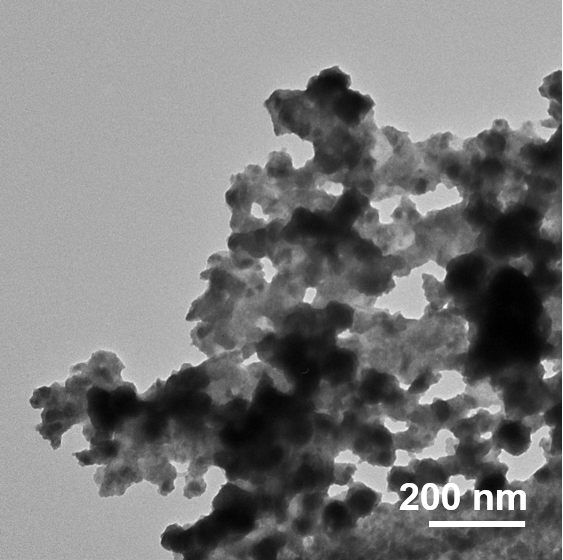


**Figure S1.** TEM image of Bi-TA.


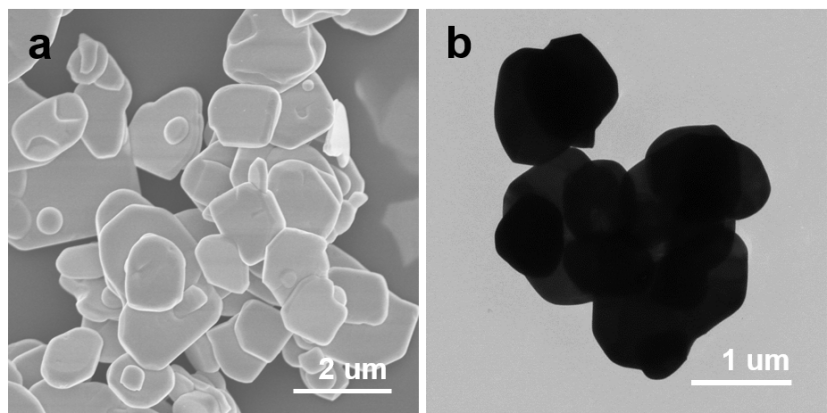


**Figure S2.** (a) SEM and (b) TEM image of BiOCl.


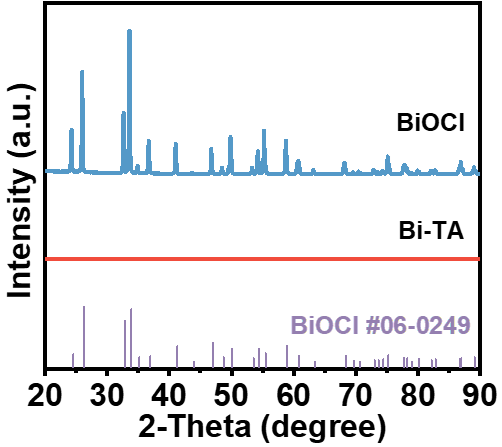


**Figure S3.** XRD patterns of Bi-TA and BiOCl.


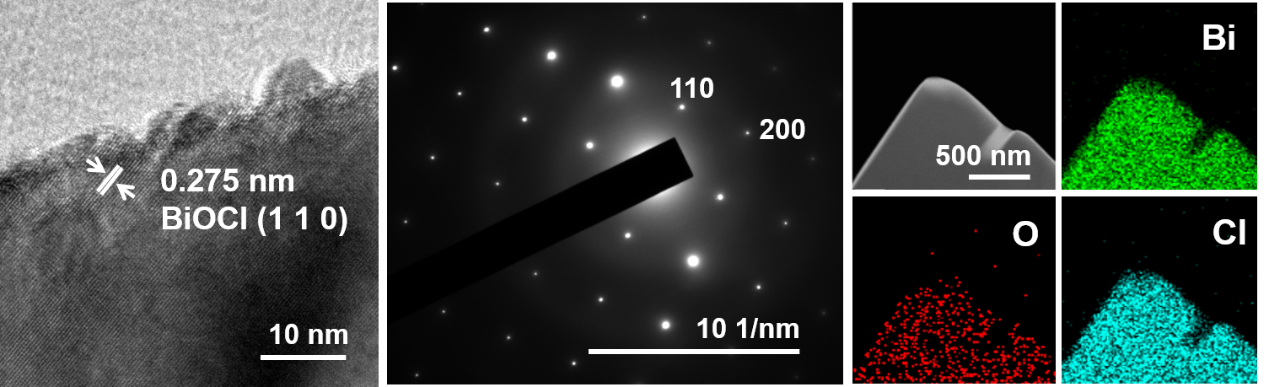


**Figure S4.** (a) HRTEM, (b) EDS and (c) SAED of BiOCl.


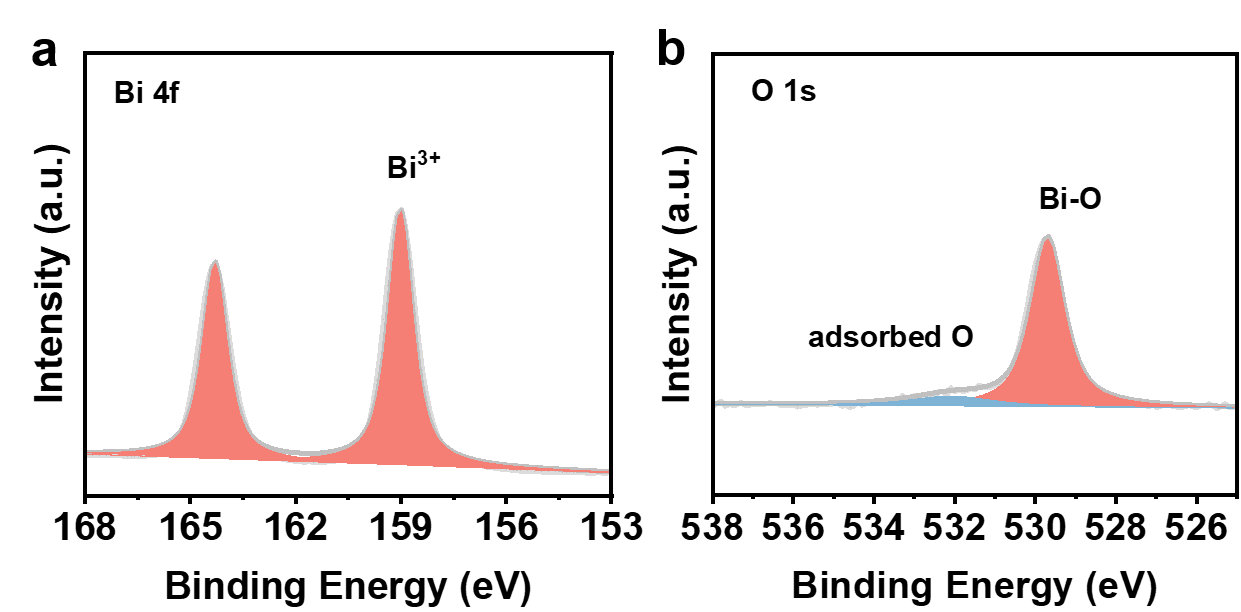


**Figure S5.** High-resolution XPS spectra of BiOCl (a) Bi 4f, (b) O 1s.


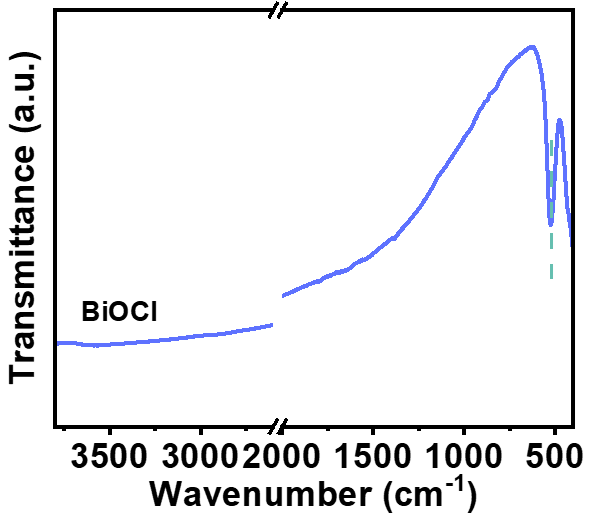


**Figure S6.** FT-IR of BiOCl.


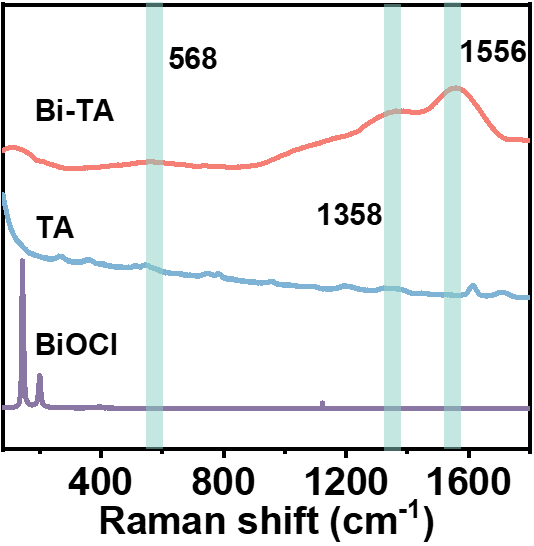


**Figure S7.** Raman spectra for Bi-TA, TA and BiOCl.


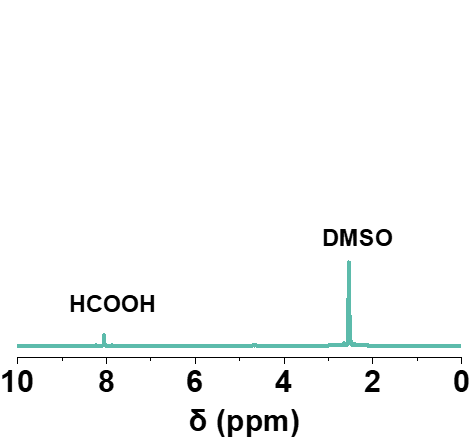


**Figure S8.** Representative NMR spectrum. The chemical shift for the internal Standard DMSO is about 2.4 and the chemical shift for formate is about 8.1.


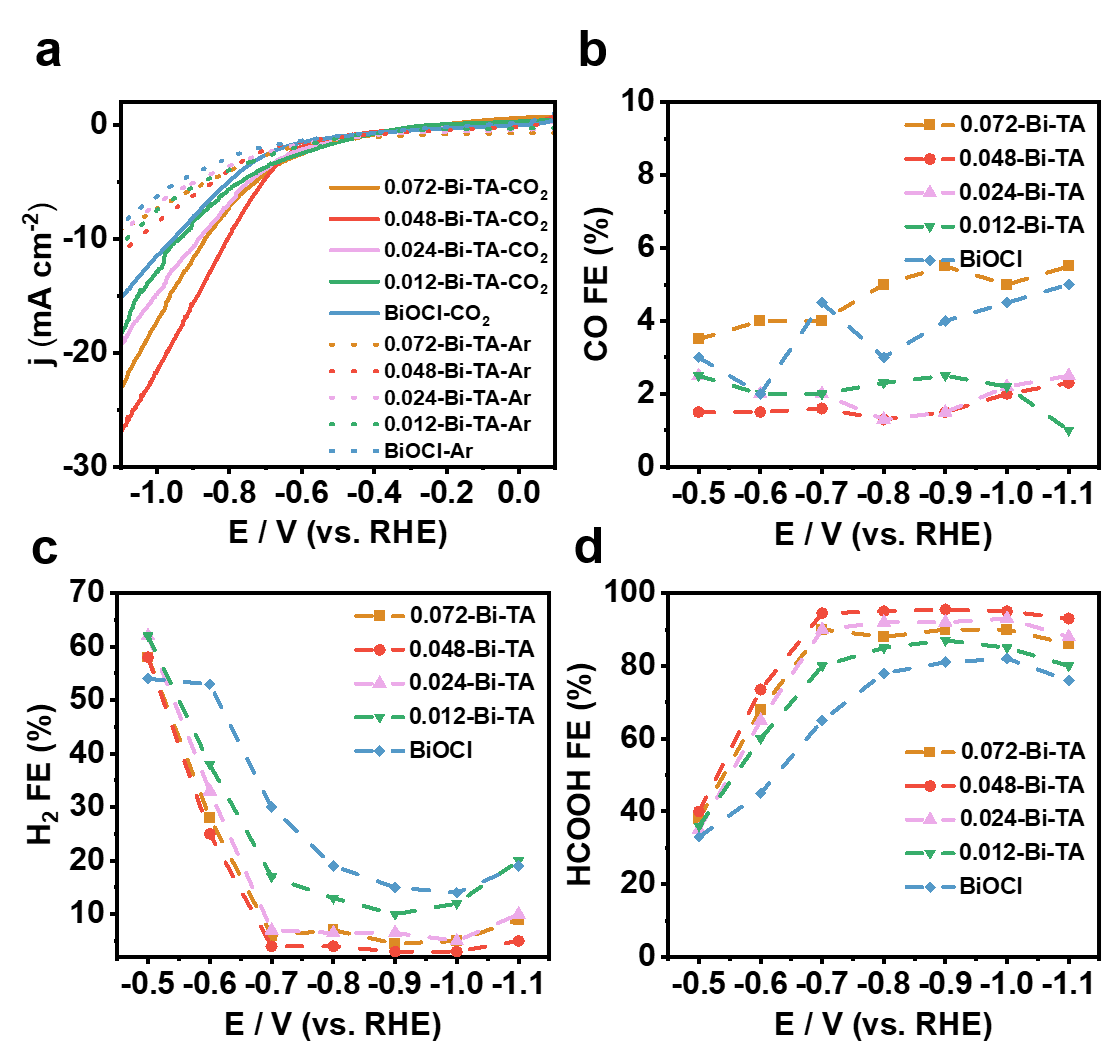


**Figure S9.** (a) LSV curves in Ar or CO_2_-saturated 0.5 M KHCO_3_, (b) CO FEs, (c) H_2_ FEs and (d) HCOOH FEs of the BiOCl and various Bi-adding TA catalysts.


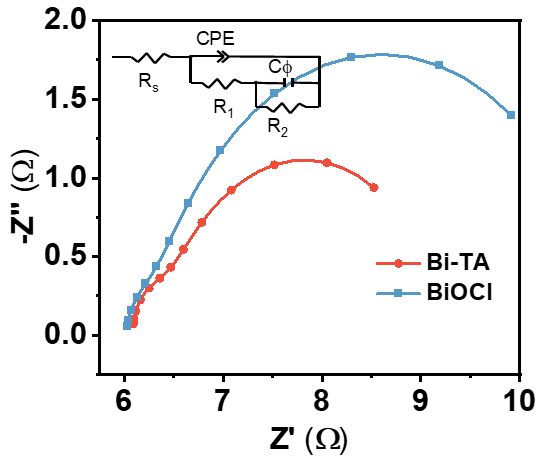


**Figure S10.** EIS Nyquist plots of the Bi-TA and BiOCl.


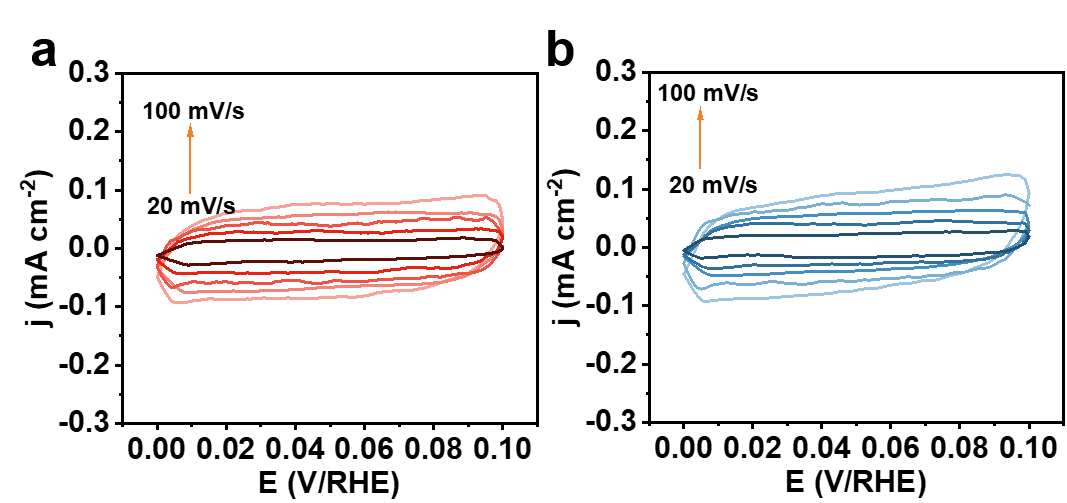


**Figure S11.** (a-b) CV curves 0.5 M KHCO_3_ electrolyte under a series of scan rates.


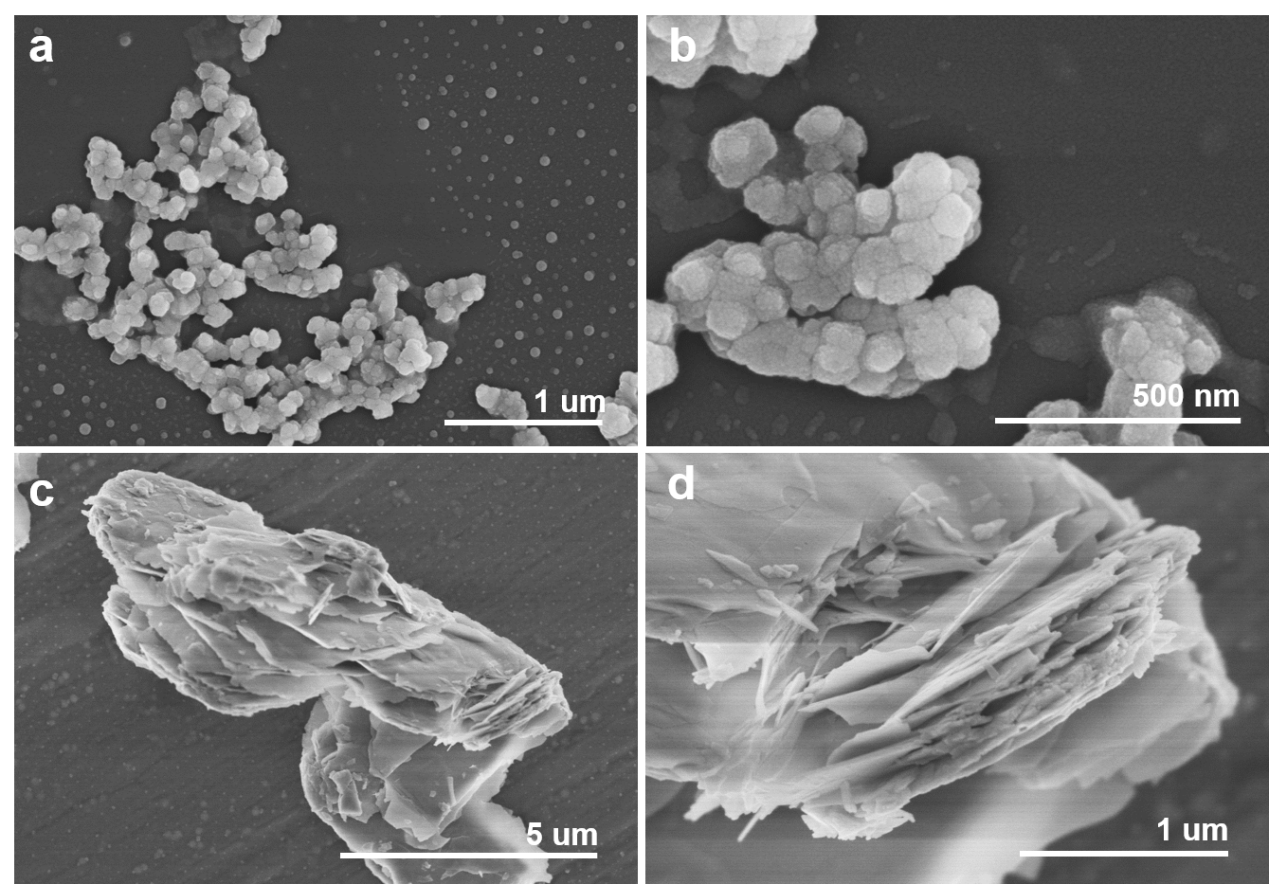


**Figure S12.** SEM of Bi-TA (a,b) and BiOCl (c,d) after soaking in 0.5 M KHCO_3_ solution.


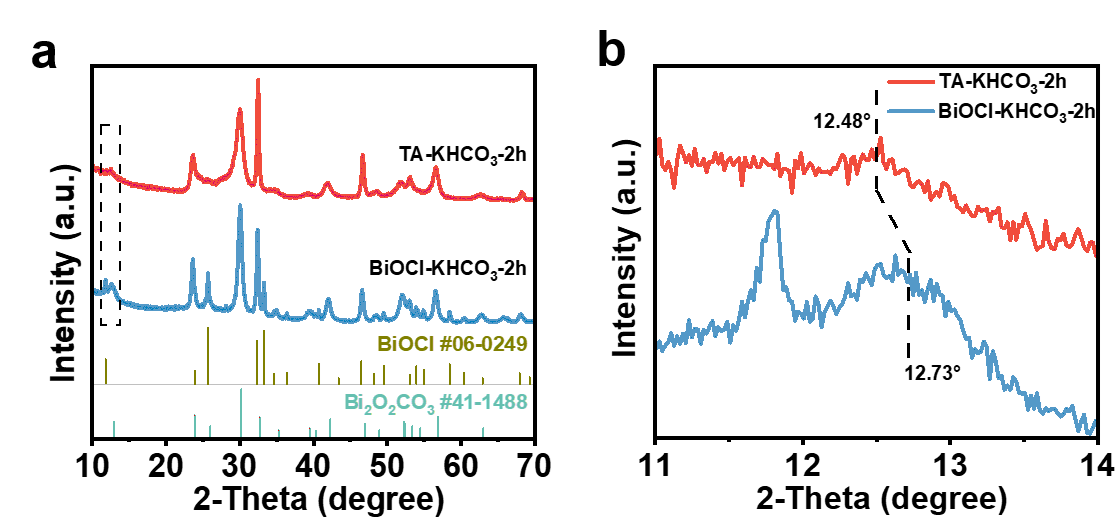


**Figure S13.** XRD of Bi-TA and BiOCl after soaking in 0.5 M KHCO_3_ solution.


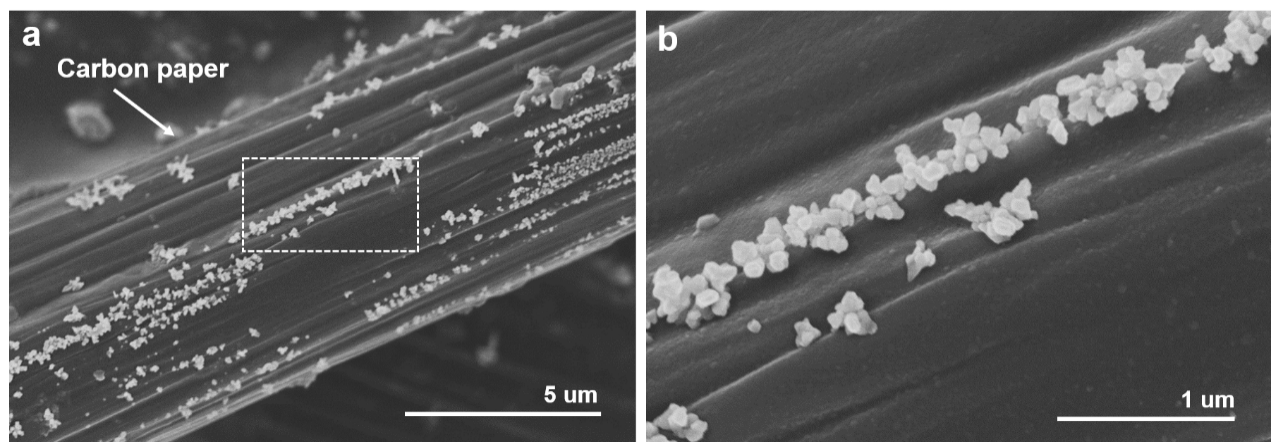


**Figure S14.** SEM images of the BiOCl after CO_2_RR tests.


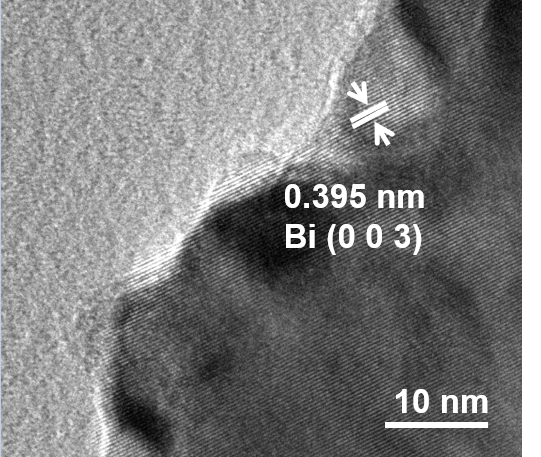


**Figure S15.** HRTEM images of the BiOCl after CO_2_RR test


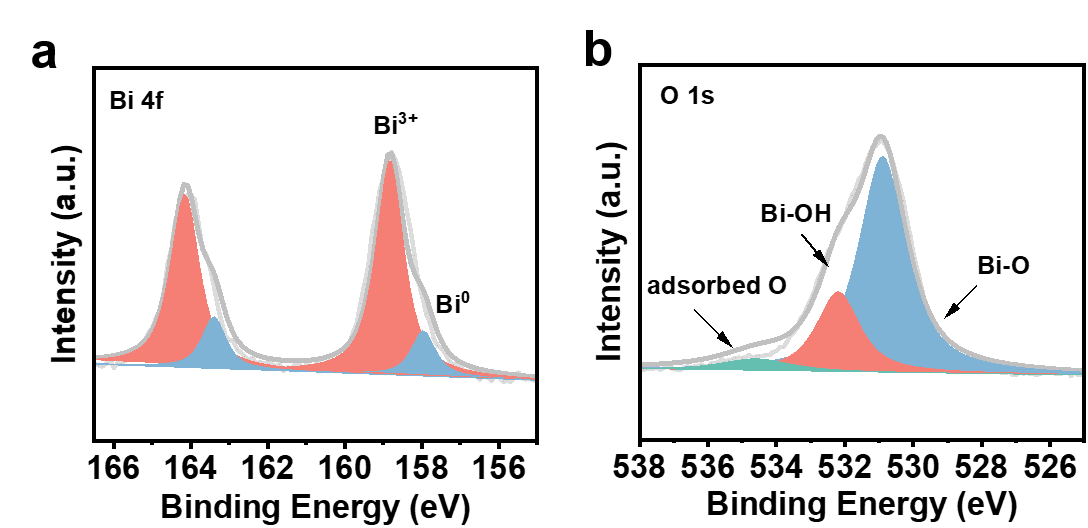


**Figure S16.** XPS spectra of Bi 4f spectrum and O 1s spectrum of BiOCl after CO_2_RR.


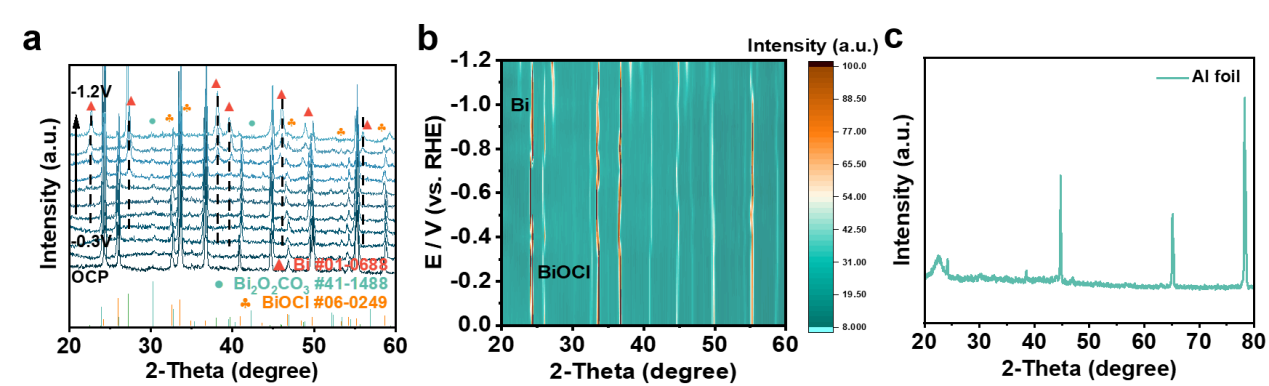


**Figure S17.** In situ XRD (a) of BiOCl recorded at various applied potentials (vs. RHE) in 0.5 M KHCO_3_ solution and (b) Corresponding contour map, (c) XRD pattern of Al foil.


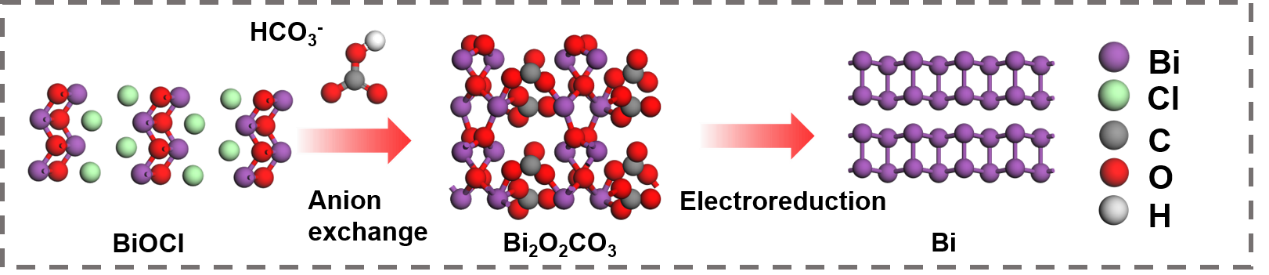


**Figure S18.** Illustration of the transformation from BiOCl to Bi.


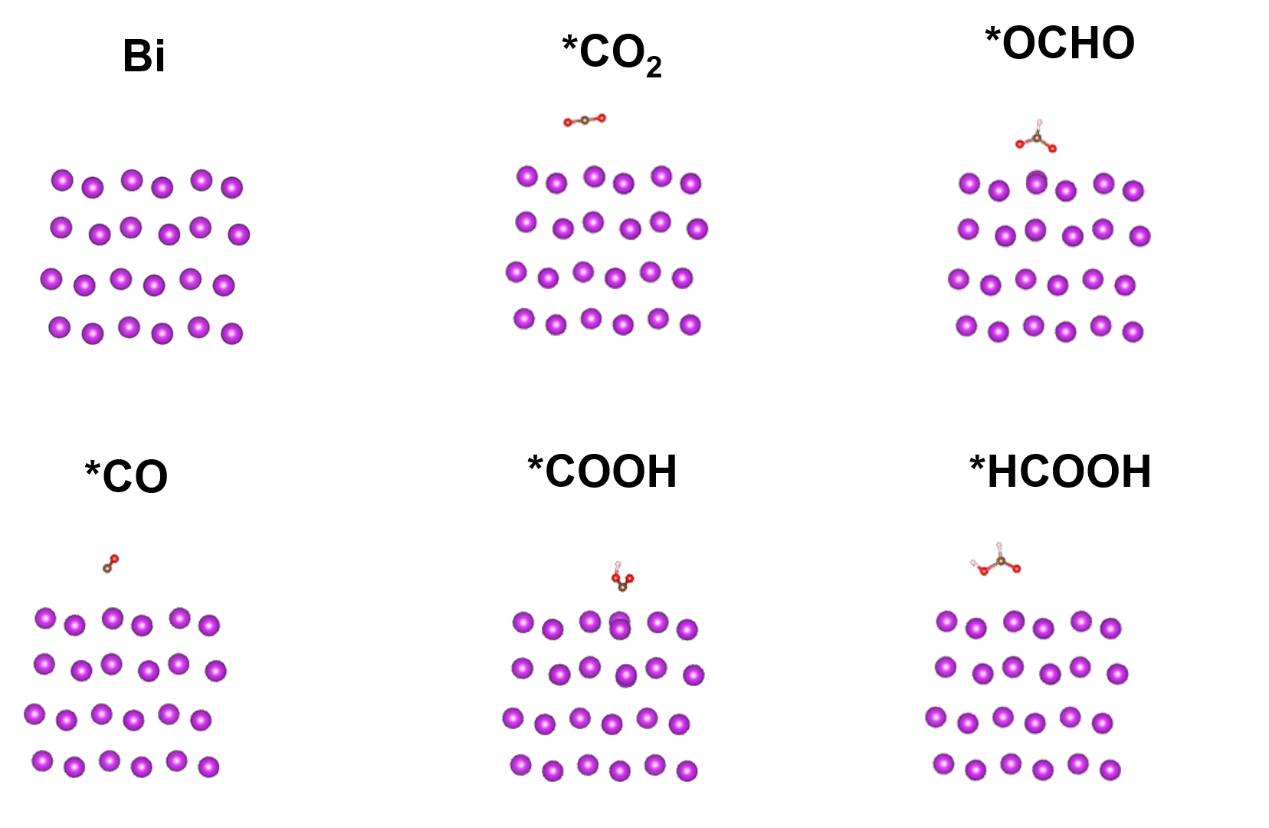


**Figure S19.** The optimized structures with adsorbed *CO_2_, OCHO*, *CO, *COOH, and *HCOOH on Bi.


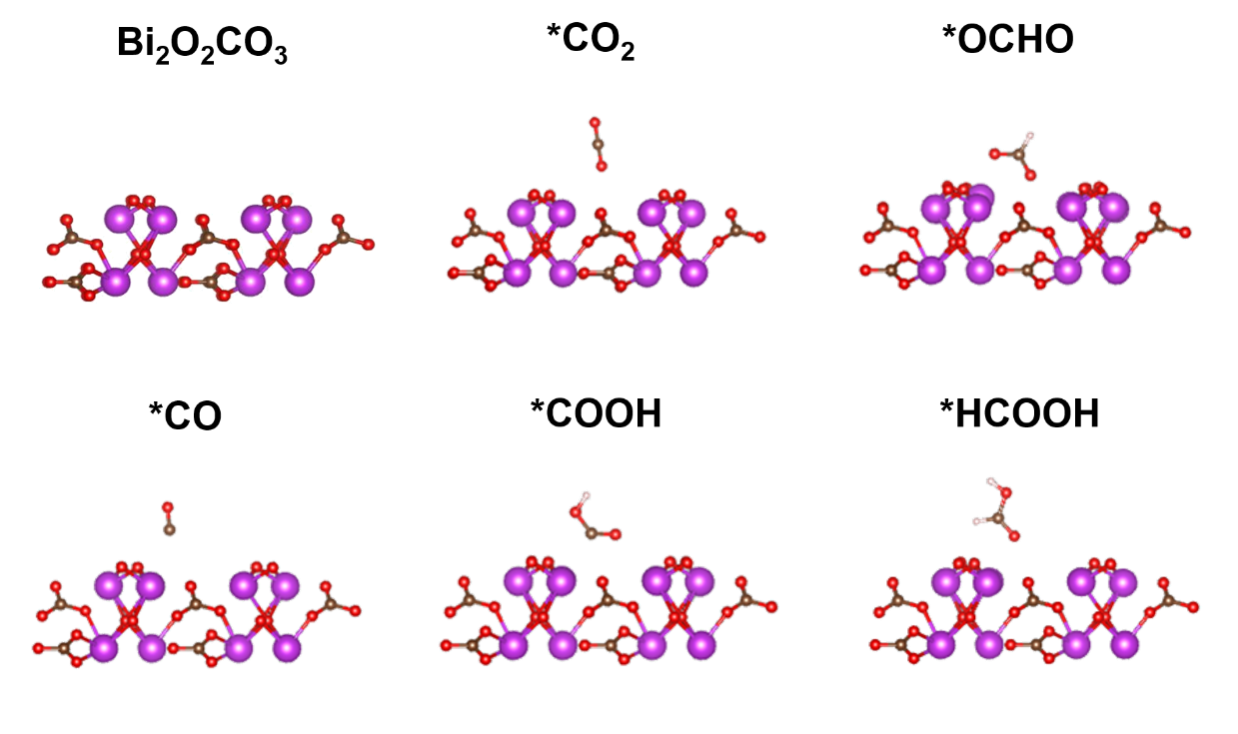


**Figure S20.** The optimized structures with adsorbed *CO_2_, OCHO*, *CO, *COOH, and *HCOOH on Bi_2_O_2_CO_3_.


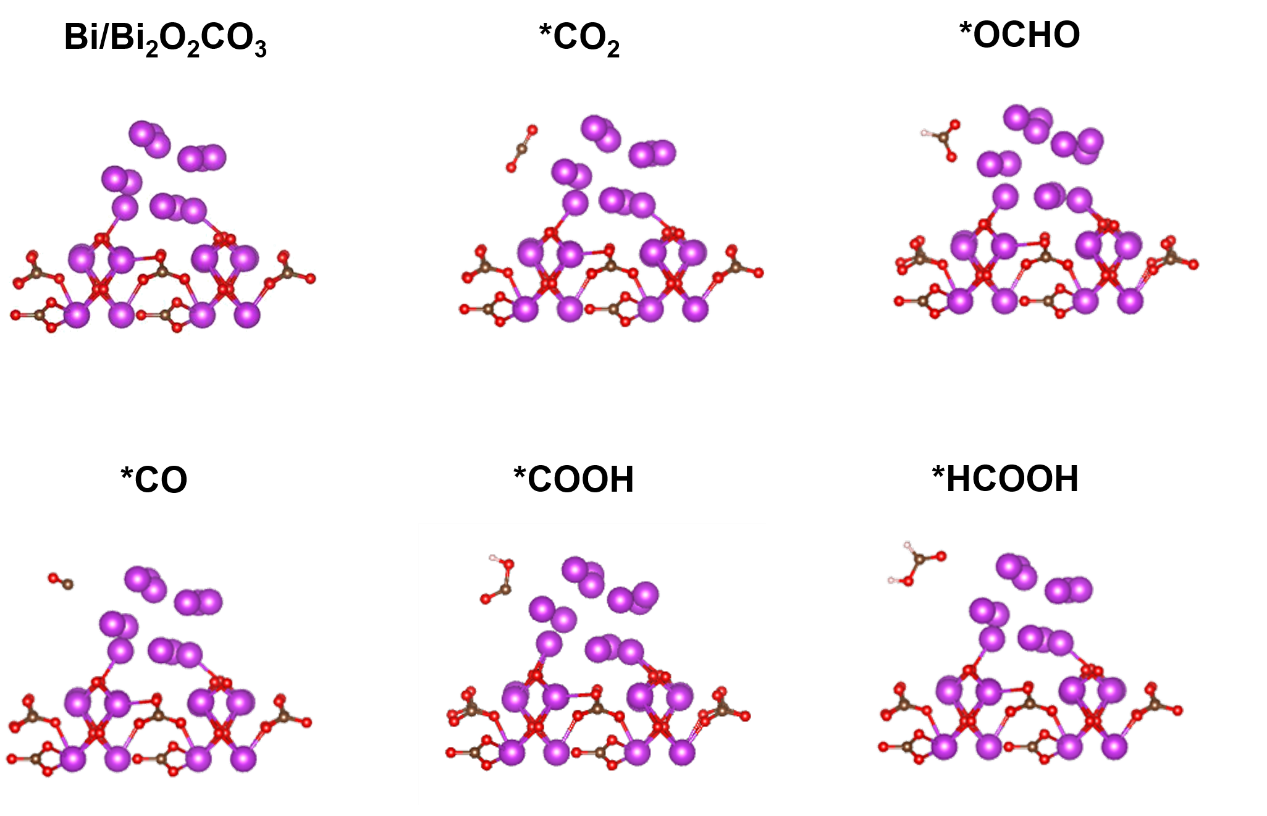


**Figure S21.** The optimized structures with adsorbed *CO_2_, OCHO*, *CO, *COOH, and *HCOOH on Bi/Bi_2_O_2_CO_3_.

.

_
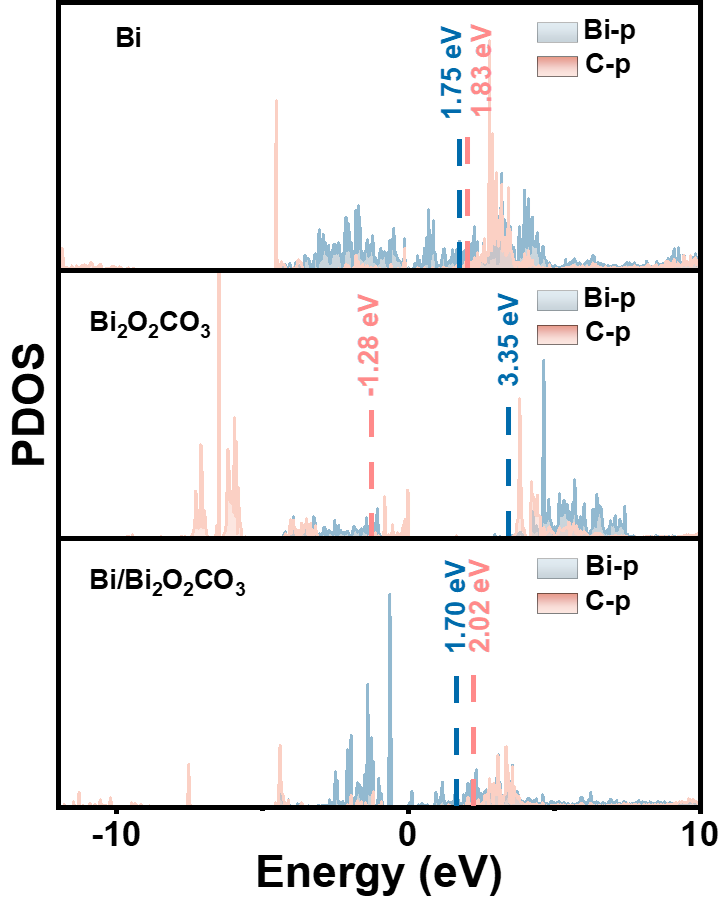
_

**Figure S22.** PDOS of the carbon atom of the *COOH intermediate and surface Bi atoms on Bi, Bi_2_O_2_CO_3,_ and Bi/Bi_2_O_2_CO_3_.


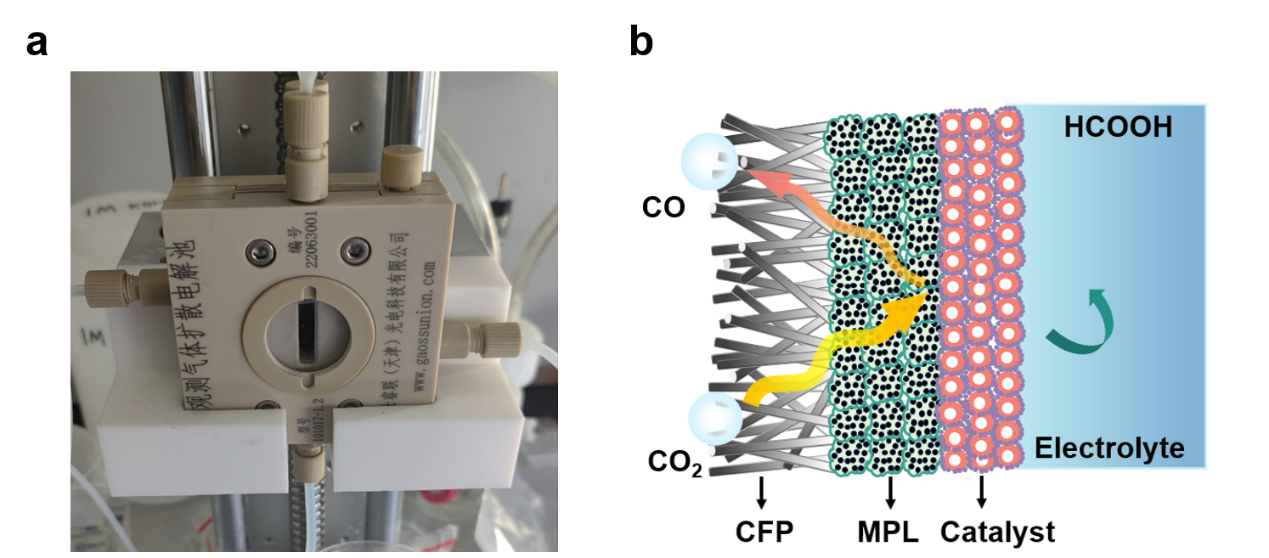


**Figure S23.** (a) Photograph of flow cell, (b) Three-phase interface in the common gas-diffusion electrode (GDE).

**Table S1**. Thermodynamic potential of electrochemical negative half-reactions for CO_2_RR.^[1]^

| Negative half-reaction | E^0^/[V vs RHE] |
| --- | --- |
| CO_2_ + 2H^+^ + 2e^−^→HCOOH | **-0.12** |
| CO_2_ + 2H^+^ + 2e^−^→CO + H_2_O | -0.10 |
| CO_2_ + 6H^+^ + 6e^−^→CH_3_OH + H_2_O | 0.03 |
| CO_2_ + 8H^+^ + 8e^−^→CH_4_ + 2H_2_O | 0.17 |
| 2CO_2_ + 12H^+^ + 12e^−^→C_2_H_4_ + 4H_2_O | 0.08 |
| 2CO_2_ + 8H^+^ + 8e^−^→CH_3_COOH + 2H_2_ | 0.11 |
| 2CO_2_ + 12H^+^ + 12e^−^→C_2_H_5_OH + 3H_2_O | 0.09 |
| 3CO_2_ + 18H^+^ + 18e^−^→C_3_H_7_OH + 5H_2_O | 0.10 |

**Table S2.** Comparison of recently reported electrocatalysts for CO_2_RR to formate.

| Catalyst | Electrolyte | Potential  (V vs. RHE) | FE_HCOOH_ | Reference |
| --- | --- | --- | --- | --- |
| **Bi-TA** | **0.5 M KHCO_3_** | **-0.9** | **96%** | **This work** |
| Bi-NRs@NCNTs | 0.1 M KHCO_3_ | -0.9 | 90.9% | ^[2]^ |
| Bi/CeOx | 0.2 M Na_2_SO_4_ | -0.9 | 90% | ^[3]^ |
| PD-Bi1 | 0.5 M KHCO_3_ | -0.9 | 91.4% | ^[4]^ |
| Bi@Sn NPs | 0.5 M KHCO_3_ | -1.1 | 91% | ^[5]^ |
| Bi-NSS | 0.1 M KHCO_3_ | -1.1 | 98.4% | ^[6]^ |
| Bi-MOF | 0.1 M NaHCO_3_ | -0.9 | 92.4% | ^[7]^ |
| Bi(btb) | 0.5 M KHCO_3_ | -1.0 | 95.3% | ^[8]^ |
| Bi_2_O_3_@C-800 | 0.5 M KHCO_3_ | -0.9 | 92% | ^[9]^ |
| Bi_2_O_3_NSs@MCCM | 0.1 M KHCO_3_ | -0.9 | 91% | ^[10]^ |

**Table S3.** Comparison of the TOF of recently reported electrocatalysts for CO_2_RR.

| Catalyst | Electrolyte | Potential  (V vs. RHE) | TOF | Reference |
| --- | --- | --- | --- | --- |
| Bi-TA | 0.5 M KHCO_3_ | -1.1 V | 1030 h^-1^ | **This work** |
| Bi@Sn NPs | 0.5 M KHCO_3_ | -1.1 V | 578.1 h^-1^ | ^[5]^ |
| [Fe_4_N(CO)_12_]^-^ | 0.1 M PBS | -1.2 V | 106±6 h^-1^ | ^[11]^ |
| Cu-CB/GDL | 0.5 M KCl | -1.1 V | 72 h^-1^ | ^[12]^ |
| Bi/Sn | 0.5 M KHCO_3_ | -1.0 V | 634.3 h^-1^ | ^[13]^ |
| Sn-Cu alloy | 0.5 M KHCO_3_ | -1.0 V | 352.5 h^-1^ | ^[14]^ |
| SnO/C | 0.5 M KHCO_3_ | -1.0 V | 373 h^-1^ | ^[15]^ |
| Sn(S)/Au | 0.1 M KHCO_3_ | -0.3 V | 951.6 h^-1^ | ^[16]^ |

**Table S4.** Optimized structural parameters.

| Materials | a b c | α β γ |
| --- | --- | --- |
| Bi | 13.78 Å 14.01 Å 30.04 Å | 90.0° 90.0° 90.0° |
| Bi_2_O_2_CO_3_ | 11.61 Å 15.04 Å 24.92 Å | 90.0° 90.0° 67.3° |
| Bi/Bi_2_O_2_CO_3_ | 11.61 Å 15.04 Å 24.92 Å | 90.0° 90.0° 67.3° |

**Table S5.** Thermodynamic quantities of gas-phase molecules (eV). E_ZPE_ and S were were taken from an report by Klinkova et al.^[17]^

|  | E_DFT_ | E_ZPE_ | TS |
| --- | --- | --- | --- |
| H_2_O | -14.22 | 0.56 | 0.67 |
| CO_2_ | -22.99 | 0.31 | 0.66 |
| H_2_ | -6.75 | 0.27 | 0.43 |
| CO | -14.77 | 0.13 | 0.67 |
| HCOOH | -29.91 | 0.89 | 1.05 |

**Table S6.** Charges transformation calculated through Bader Charge analysis.

| Adsorbates | Surface models | | |
| --- | --- | --- | --- |
|  | Bi | Bi_2_O_2_CO_3_ | Bi/Bi_2_O_2_CO_3_ |
| *COOH | 0.44 | 0.3 | 0.41 |
| OCHO* | 0.67 | 0.49 | 0.69 |

**References**

[1] J. Li, P. Yang, X. Li, C. Jiang, J. Yun, W. Yan, K. Liu, H. J. Fan, S. W. Lee, *ACS Energy Lett.* **2022**, *8*, 1-8.

[2] W. Zhang, S. Yang, M. Jiang, Y. Hu, C. Hu, X. Zhang, Z. Jin, *Nano Lett.* **2021**, *21*, 2650-2657.

[3] Y.-X. Duan, Y.-T. Zhou, Z. Yu, D.-X. Liu, Z. Wen, J.-M. Yan, Q. Jiang, *Angew. Chem. Int. Ed.* **2021**, *60*, 8798-8802.

[4] Y. Wang, Y. Li, J. Liu, C. Dong, C. Xiao, L. Cheng, H. Jiang, H. Jiang, C. Li, *Angew. Chem. Int. Ed.* **2021**, *60*, 7681-7685.

[5] Y. Xing, X. Kong, X. Guo, Y. Liu, Q. Li, Y. Zhang, Y. Sheng, X. Yang, Z. Geng, J. Zeng, *Adv. Sci.* **2020**, *7*, 1902989.

[6] F.-L. Meng, Q. Zhang, Y.-X. Duan, K.-H. Liu, X.-B. Zhang, *Chin. J. Chem.* **2020**, *38*, 1752-1756.

[7] F. Li, G. H. Gu, C. Choi, P. Kolla, S. Hong, T.-S. Wu, Y.-L. Soo, J. Masa, S. Mukerjee, Y. Jung, J. Qiu, Z. Sun, *Appl. Catal. B-Environ.* **2020**, *277*, 119241.

[8] J. Yu, H. Liu, S. Song, Y. Wang, P. Tsiakaras, *Appl. Catal. A.* **2017**, *545*, 159-166.

[9] P. Lamagni, M. Miola, J. Catalano, M. S. Hvid, M. A. H. Mamakhel, M. Christensen, M. R. Madsen, H. S. Jeppesen, X.-M. Hu, K. Daasbjerg, T. Skrydstrup, N. Lock, *Adv. Funct. Mater.* **2020**, *30*, 1910408.

[10] P. Deng, F. Yang, Z. Wang, S. Chen, Y. Zhou, S. Zaman, B. Y. Xia, *Angew. Chem. Int. Ed.* **2020**, *59*, 10807-10813.

[11] A. Taheri, E. J. Thompson, J. C. Fettinger, L. A. Berben, *ACS Catal.* **2015**, *5*, 7140-7151.

[12] C. Ampelli, C. Genovese, B. C. Marepally, G. Papanikolaou, S. Perathoner, G. Centi, *Faraday Discuss.* **2015**, *183*, 125-145.

[13] Z. Li, Y. Feng, Y. Li, X. Chen, N. Li, W. He, J. Liu, *Chem. Eng. J.* **2022**, *428*, 130901.

[14] J. Wang, J. Zou, X. Hu, S. Ning, X. Wang, X. Kang, S. Chen, *J. Mater. Chem. A* **2019**, *7*, 27514-27521.

[15] J. Gu, F. Heroguel, J. Luterbacher, X. Hu, *Angew. Chem. Int. Ed.* **2018**, *57*, 2943-2947.

[16] X. Zheng, P. De Luna, F. P. G. de Arquer, B. Zhang, N. Becknell, M. B. Ross, Y. Li, M. N. Banis, Y. Li, M. Liu, O. Voznyy, D. Cao Thang, T. Zhuang, P. Stadler, Y. Cui, X. Du, P. Yang, E. H. Sargent, *Joule* **2017**, *1*, 794-805.

[17] A. Klinkova, P. De Luna, C.-T. Dinh, O. Voznyy, E. M. Larin, E. Kumacheva, E. H. Sargent, *ACS Catal.* **2016**, *6*, 8115-8120.
